# English-Persian cross-language plagiarism detection: Multilingual deep learning approach

**DOI:** 10.1371/journal.pone.0354459

**Published:** 2026-09-30

**Authors:** Sara Ansar, Marzieh Zarinbal, Azadeh Mohebi

**Affiliations:** 1 Department of Industrial Engineering and Management Systems, Amirkabir University of Technology, Tehran, Iran; 2 Information Technology Research Department, Iranian Research Institute for Information Science and Technology (IranDoc), Tehran, Iran; Iuliu Hațieganu University of Medicine and Pharmacy: Universitatea de Medicina si Farmacie Iuliu Hatieganu, ROMANIA

## Abstract

Cross-lingual plagiarism detection (CLPD) remains a critical challenge in Natural Language Processing, particularly for low-resource language pairs where translation and paraphrasing obscure source materials. This study introduces a robust framework for Persian-English CLPD by integrating state-of-the-art sentence representations with optimized machine learning classifiers. We utilized the BAAI/bge-m3 model to generate high-dimensional embeddings, employing cosine similarity to measure contrastive linguistic distances. To evaluate classification performance, we compared various architectures, including XGBoost, LSTM, and Logistic Regression (LR), across three categories: Exact, Semi-Exact, and Different. While deep learning and ensemble methods were initially considered, experimental results on an expanded dataset demonstrated that a fine-tuned LR model provided superior stability and generalization. The optimized LR approach significantly increased classification accuracy from 89% to 96% (p < 0.05). These findings suggest that high-quality embeddings can enhance detection performance in low-resource contexts without the computational overhead of complex architectures. To ensure transparency and reproducibility, our dataset and source code are available on GitHub.

## Introduction

The rapid advancement of machine translation technologies and multilingual large language models (LLMs) has significantly increased the risk of cross-lingual plagiarism, where translated text is presented as original without proper attribution [1]. Compared with monolingual plagiarism detection, cross-lingual detection is more challenging because it requires identifying semantic equivalence across languages with different lexical, syntactic, and morphological structures. As a result, it has become an important area of research for maintaining academic integrity in multilingual environments.

Over the past decade, various approaches have been proposed to address this problem. Early methods relied on machine translation (MT) to convert texts into a single language, followed by traditional monolingual plagiarism detection. More recent studies have focused on cross-lingual alignment techniques, multilingual embeddings, and transformer-based models, which improve semantic matching across languages. However, these methods still depend heavily on high-quality resources and benchmark datasets, and their performance varies across language pairs.

Despite these advances, important challenges remain, especially for low-resource languages such as Persian. The lack of large benchmark datasets and the presence of complex morphological and syntactic structures make reliable detection difficult. In addition, AI-assisted translation tools now generate highly fluent texts that are harder to distinguish from original writing, further increasing the difficulty of detection.

To address these challenges, this study proposes a deep learning framework for Persian–English cross-lingual plagiarism detection. The framework combines multilingual sentence embeddings with supervised classifiers to improve semantic discrimination across plagiarism levels. An AI-assisted benchmark dataset is constructed to reflect modern translation characteristics and varying degrees of similarity. The proposed method is evaluated through comprehensive experiments and statistical analysis.

## Literature review

This section reviews previous studies in detecting plagiarism and categorizes them to identify research gaps. Machine translation-based analysis and cross-lingual alignment-based similarity analysis (CL-ASA) is the most widely used methods in this field.

### Machine translation-based analysis

The most straightforward approach to cross-lingual plagiarism detection involves leveraging Machine Translation (MT) to reduce the problem to a monolingual one. While the accessibility of translation tools makes this method popular, its reliability is inherently tied to the performance of the underlying MT system. Early implementations, such as the work by Kasprzak and Brandejs [2] and Pataki [3,4], demonstrated that while large-scale detection is feasible, issues like poor translation quality in complex academic texts and reliance on public APIs often limit scalability and accuracy.

The literature also highlights a recurring struggle with computational costs and language-specific nuances. For instance, while Kent and Salim [5] utilized Google’s infrastructure for English-Arabic detection, their reliance on k-gram matching proved computationally expensive. To mitigate these efficiency and accuracy gaps, researchers have integrated more sophisticated layers; Alzahrani and Aljuaid [6] and Safi-Esfahani et al. [7] successfully employed deep learning and semantic analysis to filter out false positives particularly in Persian and Arabic contexts. However, as noted by Ferrero et al. [8] and Pinto et al. [9], MT-based models still face a “performance ceiling” when dealing with low-resource languages or complex syntactic structures [10, 11], where translation errors directly degrade the recall of the entire detection system.

### Cross-lingual alignment-based similarity analysis

Beyond translation-based methods, CL-ASA offers a more direct route by aligning equivalent sentences or phrases across different languages. This technique, pioneered by Barrón-Cedeño et al. [12], minimizes the dependency on machine translation quality by focusing on structural and feature-based alignments. To tackle the nuances of heavily paraphrased content, Franco-Salvador et al. [13] later integrated knowledge graphs into this framework, significantly sharpening the model's semantic depth. Practical applications, such as those explored by Gupta et al. [10], confirm that this approach is particularly adept at spotting translated plagiarism in diverse pairs like Hindi and English. Ultimately, as noted by Potthast et al. [14], the true strength of CL-ASA lies in its capacity to detect long-range reuse within dense academic texts a task where traditional word-for-word methods often fail.

### Advanced approaches

To overcome the inherent limitations of machine translation (MT) and capture complex linguistic variations, recent research has shifted toward deep learning (DL), word embeddings, and sophisticated semantic analyses. The primary objective is to enhance detection accuracy while optimizing processing time for languages with significant structural disparities.

### Persian and multilingual contexts

In the context of Persian plagiarism detection, Gharavi et al. [15,16] and Pakniat and Mohebi [1] have demonstrated that converting text into numerical vectors and employing DL frameworks can effectively identify word-order changes and synonym substitutions, achieving accuracies up to 95.9%. These methods often utilize sentence-level comparisons and cosine similarity, although some trade-offs exist regarding the retention of word-order information. To further refine this, Roostaee et al. [17] introduced a two-level alignment approach integrating syntactic and semantic features via graph-based word representations, ensuring stable performance across diverse datasets.

### Arabic and cross-lingual benchmarks

Arabic-English plagiarism detection has also seen significant advancement. Ezzikouri et al. [18] and Alotaibi et al. [19] evaluated various methods, including cross-lingual character n-grams (CL-CNG) and multilingual encoders, finding that while MT-based analysis remains accurate, embedding-based techniques provide a more efficient alternative for distant language pairs. Furthermore, the development of benchmark datasets, such as the JUPlag system [20], and the Urdu-English dataset by Haneef et al. [21], has provided essential resources for training and testing these sophisticated models.

### Hybrid and scalable systems

Hybrid systems that combine statistical methods with DL have proven particularly effective for large-scale detection. Bakhteev et al. [22] and Gang et al. [23] proposed multi-stage processes identifying candidates first, then analyzing fragments, while utilizing knowledge distillation to improve efficiency. Similarly, Corezola et al. [24] and Al-Jibory et al. [25] designed comprehensive systems incorporating sliding-window techniques and hybrid feature extraction (textual and non-textual) to generate detailed similarity reports.

Despite these technical strides, systematic reviews by Botto-Tobar et al. [26] and Tlitova et al. [27] highlight a persistent lack of specialized tools and standardized methodologies across many studies. These researchers emphasize the need for more robust detection methods as global academic publishing continues to expand across diverse language pairs (e.g., Russian-English), necessitating systems that can navigate complex ethical concerns and varied representation types

### Research gap

Table 1 summarizes prior studies. Regarding the Geographic and Linguistic Gap, most studies focus on European or Arabic-English pairs [6]. While work exists for Persian [16], there is a lack of high-performance frameworks leveraging recent multilingual deep learning specifically for this pair. In terms of Semantic Preservation Issues, existing MT-based methods [7] often fail to capture subtle nuances or struggle with scalability. Even with semantic analysis [5], dependency on traditional translation remains a bottleneck for complex academic texts.

**Table 1:** Comparative Table of Related Works and This Research.

Reference	Translation	Translation + Paraphrasing	AI-based Translation	Granularity	Deep Learning Combination	Text Labeling	Similarity Analysis	Language
[24]	✓		✓	Word	✓			Persian
[13]	✓			Word	✓			German
[15]			✓	Word	✓		✓	Persian
[7]		✓		Word				Persian
[8]	✓	✓		Word		✓		French
[18]	✓		✓	Word	✓	✓		Arabic
[1]		✓		Sentence	✓		✓	Persian
[21]	✓			Word		✓		Urdu
[17]	✓			Word	✓	✓		English
[20]	✓			Word	✓			Arabic
[27]	✓			Word	✓	✓		Persian
[25]	✓			Word	✓	✓		Arabic
[6]	✓			Sentence	✓			Arabic
[22]	✓			Word	✓			European
This Study		✓	✓	Sentence	✓	✓	✓	Persian

Furthermore, addressing the problem of Overlooking Obfuscation, much research focuses on verbatim translation, neglecting rewritten or paraphrased plagiarism. Current alignment models often require extensive corpora or complex graphs that are not optimized for low-resource Persian-English data. Concerning Representation Limitations, many approaches rely on n-grams [28] or shallow embeddings that ignore word order. This research addresses this by using BGE-M3 encoders to process both languages in a joint semantic space. Finally, regarding the Lack of Statistical Rigor, studies often report high accuracy but lack rigorous statistical significance testing or large-scale robustness checks. There is a critical need for validated tools that adapt to evolving plagiarism strategies.

## Materials & methods

This study follows a methodological framework that outlines the key steps undertaken in the research process. The dataset consists of the abstracts of Persian scientific documents along with their English translations, and to enhance the diversity of the dataset, two versions, semi‑exact and different, were generated by applying semantic and structural transformations to the original abstracts.

For text representation and similarity measurement, the BAAI/BGE‑M3 model is utilized to convert abstracts into vector representations, after which the cosine similarity between Persian and English abstracts is calculated to assess their resemblance. Based on the obtained similarity scores, the abstracts are labeled into predefined categories, and the dataset is subsequently divided into training and testing subsets.

In the classification phase, various classification algorithms are applied and evaluated using accuracy, recall, and F1‑score to determine their effectiveness. Finally, the best‑performing models undergo fine‑tuning and further evaluation to enhance their performance. By following this methodology, we ensure a robust approach to detecting cross‑lingual plagiarism between Persian and English texts. Fig 1 visually represents the framework, from data preparation to model evaluation.

**Fig 1:**
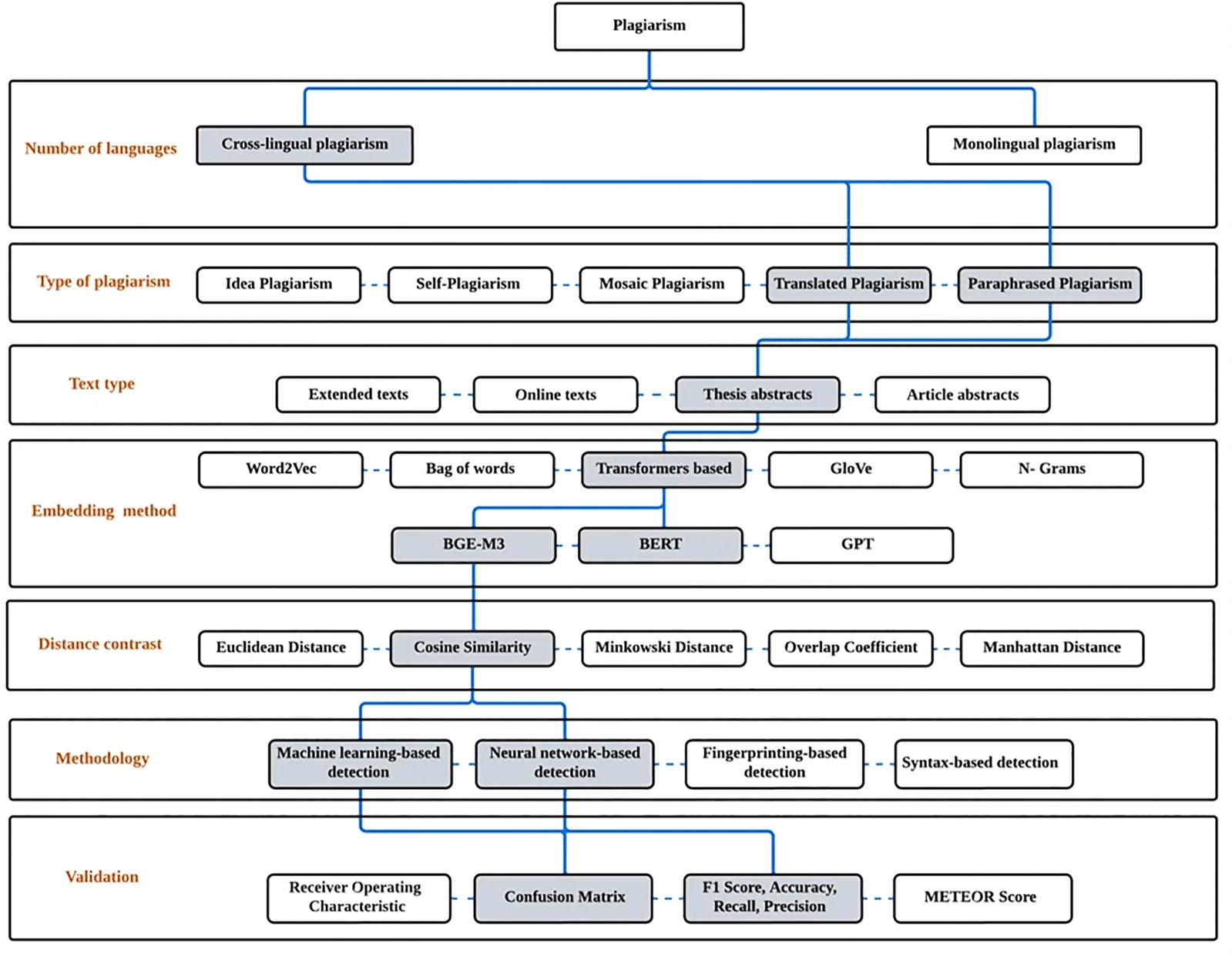
The Framework.

### Dataset

The empirical foundation of this study comprises a curated dataset of 1,000 academic abstracts sourced from the Iranian Research Institute for Information Science and Technology (IranDoc). To facilitate the investigation of cross-lingual plagiarism detection between Persian and English, the original Persian abstracts underwent a systematic translation and modification pipeline. While traditional methodologies have frequently utilized conventional machine translation (MT) engines, this research intentionally employed Microsoft Copilot for the translation phase. This selection was motivated by two primary factors: first, the advanced capability of large language models (LLMs) to preserve nuanced semantic structures in technical discourse [29]; and second, the necessity for the detection model to remain robust against modern, AI-mediated translation artifacts that researchers may encounter in contemporary academic environments.

For preparing the dataset, these English translated texts were then modified and categorized into three groups: Exact, Semi-Exact, and Different, as summarized in Table 2.

**Table 2:** Overview of the Dataset Classes and Generation Methods.

Class	Count	Generation Method	Notes
Exact	334	Direct translation	Persian abstracts translated via Copilot without modifications
Semi-Exact	334	Synonym substitution + sentence reordering	NLTK and WordNet were used; manual supervision ensured semantic consistency [30]; [31].
Different	332	Addition of external sentences	Built upon Semi-Exact texts to simulate complex plagiarism patterns, mixing new content to appear original

The Exact category contains the translated versions of Persian abstracts without any modifications. In the Semi-Exact category, the translated text was slightly paraphrased to mimic concealed plagiarism. The following two paraphrasing techniques were applied:

To systematically augment the text, two primary paraphrasing techniques were employed. First, synonym substitution was conducted by replacing specific words with their appropriate synonyms utilizing the NLTK library and the WordNet database [30]. Second, sentence reordering was applied to alter sentence structures while carefully preserving the overall contextual meaning. Since structural modifications could potentially impact semantic coherence, manual supervision was subsequently performed to guarantee the logical consistency of the generated text [31].

The Different category was generated by further modifying the Semi-Exact texts through the addition of external sentences from unrelated sources. This approach simulated complex plagiarism, where a plagiarized text is rewritten and mixed with new content to appear original. This method enabled the model to learn sophisticated plagiarism patterns beyond superficial textual similarity, capturing deeper semantic similarities, including conceptual or ideational theft.

Two human annotators independently labeled all paraphrased texts, and manual supervision ensured high agreement. Finally, basic preprocessing steps, including tokenization, lowercasing, and punctuation removal, were applied to all texts to standardize the dataset before embedding generation.

### Embedding method

In this step, the textual dataset is converted into numerical vector representations. These vectors provide a numerical representation of sentences, enabling models to capture the semantic and syntactic features of the text. Unlike word-level representations, a sentence-level approach allows for a more precise evaluation of meaning and structural variations. This is particularly important in cross-lingual plagiarism detection, where minor changes in wording can significantly alter the interpretation of a sentence. By focusing on entire sentences rather than individual words, this method enhances the accuracy of similarity detection and ensures a more reliable assessment of content transformation.

This method facilitates the extraction of similarities between Persian and English texts through vector-based computations. Common sentence representation techniques include Word2Vec, GloVe, FastText, and BERT [32].

In this study, we used two embedding models, BERT and BGE-M3-Embeddings, to generate sentence vectors, enabling numerical representation of sentences for similarity measurement. BERT is widely recognized for its deep contextual understanding, while BGE-M3-Embeddings is specifically designed for tasks involving cross-lingual similarity. By leveraging both models, we aimed to obtain rich vector representations that capture semantic and syntactic variations between Persian and English abstracts.

The Bidirectional Encoder Representations from Transformers (BERT) model, introduced by Vaswani et al. [32], is a widely used method for generating contextualized embeddings. Built on the transformer architecture, BERT employs multi-head self-attention mechanisms to process input sequences bidirectionally, ensuring a deeper understanding of linguistic structures. The input text undergoes tokenization using a specialized word piece tokenizer, which splits words into sub-word units. These tokens are then mapped to high-dimensional vector representations through multiple transformer layers. The final output provides contextual embeddings that enhance natural language processing tasks, particularly in cross-lingual and semantic understanding applications.

The M3-Embedding model was introduced by Chen et al. [33] in 2024, featuring multilingual, multi-functional, and multi-granular capabilities. It supports semantic retrieval in over 100 languages and performs dense, multi-vector, and sparse retrieval tasks. The model handles input sequences from short sentences to documents up to 8192 tokens. The BGE-M3 model is built on the transformer architecture. Input tokens pass through layers of self-attention and feed-forward networks, mapping tokens to high-dimensional spaces. Final representations are aggregated into 1024-dimensional dense vectors. This embedding model optimizes multilingual text representation and maximizes GPU utilization, crucial for high-performance retrieval tasks [33].

To determine the most suitable embedding model for this study, we employed Cosine similarity to analyze the semantic alignment of Persian-English text pairs. Unlike distance-based metrics such as Euclidean distance, Cosine similarity measures the angle between vectors, making it well-suited for high-dimensional text representations. By leveraging this method, the semantic similarity between two texts can be quantified based on the angle between their corresponding vectors (a smaller angle (closer to 0 degrees) indicates a higher degree of similarity [34]. Mathematically, cosine similarity between two vectors X = (𝑥_1_, 𝑥_2_,  ..., 𝑥_n_) and Y = (𝑦_1_,𝑦_2_,  ..., 𝑦_n_) in an n-dimensional space is computed as Eq. (1):


$$\begin{array}{c} Cosine\;similarity(X,Y)=\frac{\sum_{i=\text{1}}^{n}x_{i}y_{i}}{\sqrt{\sum_{i=\text{1}}^{n}x_{i}^{\text{2}}*\sqrt{\sum_{i=\text{1}}^{n}y_{i}^{\text{2}}}}} \end{array}$$ (1)

Using BERT and BGE-M3-Embeddings, numerical representations of Persian-English text pairs are generated, and the similarities are computed. Then the contrastive distance between each Persian abstract and its corresponding English counterpart is calculated using Eq. (2):


$$\begin{array}{c} D_{\text{contrast}}=\text{1}-\frac{AB}{\left\|A\right\|\left\|B\right\|} \end{array}$$ (2)

where, A is the embedding of the Persian abstract and B is the embedding of the English abstract.

Contrastive Distance in the context of Large Language Models (LLMs) and representation learning is a metric used to evaluate the proximity of semantic embeddings within a high-dimensional vector space. It operates on the principle of distance-based discrimination, where the model is tasked with minimizing the distance between ‘positive’ pairs (e.g., a Persian abstract and its accurate English translation) while simultaneously maximizing the gap for ‘negative’ or dissimilar pairs (e.g., unrelated or heavily manipulated texts) [35].

### Evaluation metrics

To evaluate model performance, we used accuracy, precision, recall, and F1-score [35]. Accuracy measures the proportion of correctly predicted instances defined as Eq. (3):


$$\begin{array}{c} Accuracy=\frac{TP+TN}{TP+TN+FP+FN} \end{array}$$ (3)

Precision measures the proportion of correctly predicted positive instances among all predicted positives, defined as Eq. (4):


$$\begin{array}{c} Precision=\frac{TP}{TP+FP} \end{array}$$ (4)

Recall measures the proportion of correctly predicted positive instances among all actual positives, defined as Eq. (5):


$$\begin{array}{c} Recall=\frac{TP}{TP+FN} \end{array}$$ (5)

F1-score is the harmonic mean of precision and recall, providing a balanced measure for imbalanced classes calculated as Eq. (6):


$$\begin{array}{c} F\text{1}-score=\frac{Precision*Recall}{Precision+Recall} \end{array}$$ (6)

These metrics were computed for each class (Exact, Semi-Exact, Different) and averaged using the macro approach to ensure fair evaluation across all categories.

### Experimental setup and classification framework

In the first stage of the experiments, the classification models were evaluated on a dataset containing 500 samples, with three predefined categories: Exact, Semi-Exact, and Different. The objective was to identify the most effective classifier for cross-lingual plagiarism detection. To this end, seven widely used machine learning and deep learning models, including Multi-Layer Perceptron (MLP), Feedforward Neural Network (FNN), Support Vector Machine (SVM), XGBoost, Recurrent Neural Network (RNN), Long Short-Term Memory (LSTM), and Logistic Regression (LR), were trained and evaluated. In this study, performance metrics are interpreted based on standard NLP benchmarks, where scores above 90% are considered strong, 80–90% as good, and 70–80% as moderate

In the second stage, the experimental analysis was extended by increasing the dataset size from 500 to 1,000 samples in order to evaluate the impact of data scale on classification performance. Using the same three predefined categories (Exact, Semi-Exact, and Different), five representative classifiers were reassessed, including Long Short-Term Memory (LSTM), Support Vector Machine (SVM), XGBoost, Recurrent Neural Network (RNN), and Logistic Regression (LR). The models were evaluated using precision, recall, and F1-score to provide a comprehensive comparison.

In the context of academic integrity, minimizing “False Negatives” (missed plagiarism) is paramount, making Recall a prioritized metric. To optimize detection across varying degrees of similarity, the framework establishes distinct coverage boundaries using specialized classifiers. For lower-bound coverage targeting exact categories, XGBoost was selected due to its superior ability to capture direct translations, achieving the highest recall of 0.84 and ensuring that verbatim theft is identified with high sensitivity. Conversely, for upper-bound coverage across different categories, Logistic Regression was utilized because of its peak performance in the most complex classes, yielding a maximum recall of 0.91 and providing a robust defense against sophisticated, multi-source plagiarism.

By focusing on these two complementary frameworks, one based on ensemble gradient boosting and the other on statistical linear weighting the study ensures a balanced and transparent detection system. This approach not only optimizes accuracy across all levels of plagiarism but also maintains computational efficiency, making it the most viable solution for the finalized detection framework.

### Fine tune and statistical validation strategies

To enhance model sensitivity to subtle cross-lingual semantic variations and improve class separation between highly similar plagiarism categories, a systematic hyperparameter fine-tuning phase was executed. For the baseline dataset configuration consisting of 500 samples, XGBoost and LSTM were selected for optimization due to their complementary structural properties.

The fine-tuning configuration for the XGBoost model focused on regularization adjustments, settling on a subsample rate of 0.4 and a colsample_bytree ratio of 0.6 to minimize potential over-parameterization.

For the baseline LSTM model, structural complexity was increased by implementing a three-layer architecture with 128 hidden units per layer to capture deeper semantic dependencies. To ensure training stability and prevent overfitting at this scale, a dropout rate of 0.5 was applied along with gradient clipping, a refined learning rate of 0.0005, and an early stopping criterion.

For the scaled-up experiments utilizing 1,000 samples, XGBoost and Logistic Regression (LR) were chosen for deeper refinement. Automated hyperparameter tuning for the scaled-up XGBoost architecture was executed via the Optuna framework to systematically search the algorithmic configuration space. For the Logistic Regression classifier, optimization focused on refining the regularization parameters to better map decision boundaries within the dense, high-dimensional embedding spaces.

To ensure that the observed performance variations between the baseline configurations and the fine-tuned models were not the result of stochastic variation or random chance, a McNemar’s test for statistical significance was performed. This non-parametric statistical test is specifically suited for comparing the paired performance of two classifiers evaluated on the same empirical dataset by analyzing the discordance between their respective correct and incorrect predictions.

## Results

### Embedding evaluation results

The comparative analysis of embedding models, as visualized in Fig 2, demonstrates a significant disparity in their capacity to represent cross-lingual semantic relationships. The BERT embeddings (Fig 2a), exhibit high intra-cluster density with negligible variance between categories. This indicates that BERT suffers from representation collapse in this context; it generates a narrow distribution of similarity scores that fails to distinguish between an exact translation and a sophisticatedly paraphrased text. Consequently, the contrastive distance in the BERT space is insufficient to provide a clear decision boundary for plagiarism detection.

**Fig 2:**
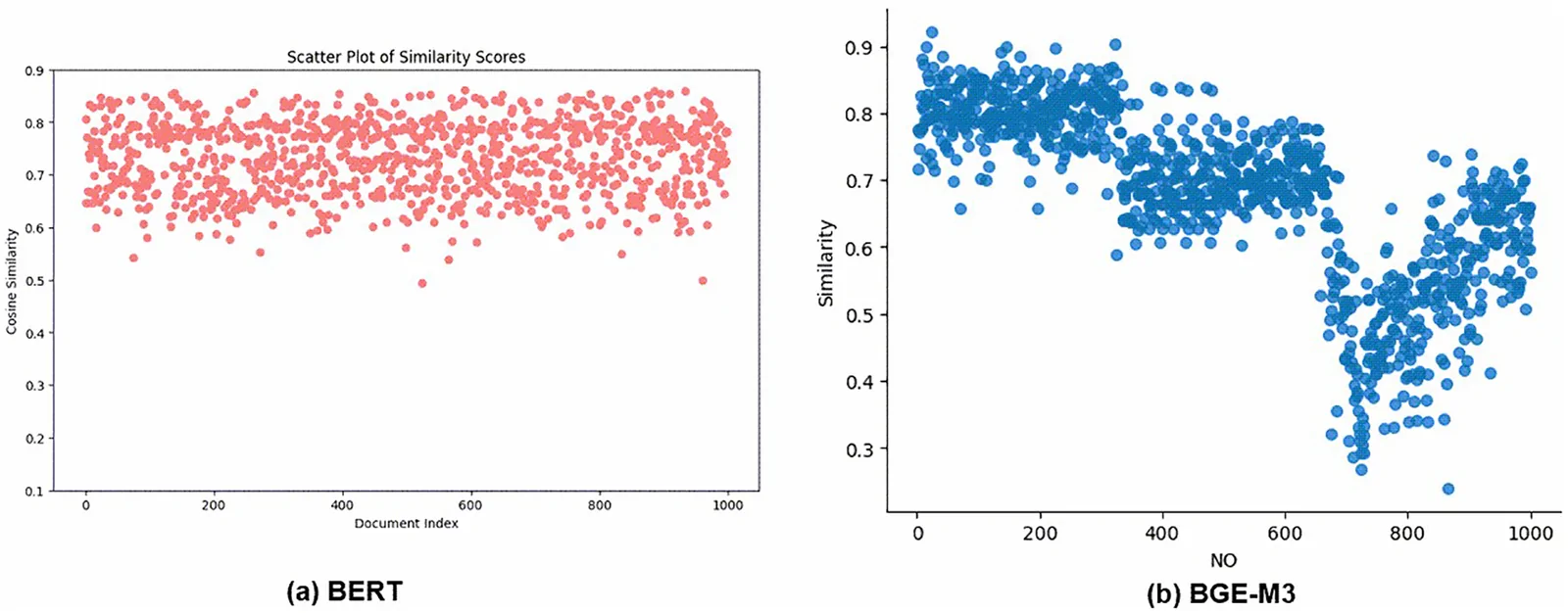
Contrastive distance of (a) BERT, (b) BGE-M3.

In contrast, the BGE-M3-Embeddings (Fig 2b) yield a robust and stratified distribution of similarity scores, spanning a wide dynamic range (approximately 24% to 92%). The effectiveness of BGE-M3 is attributed to its contrastive learning architecture, which is specifically optimized to maximize the contrastive distance between semantically distinct pairs.

As illustrated, BGE-M3 successfully isolates the three translation categories (Exact, Semi-Exact, and Different) into observable clusters. This high discriminative resolution proves that BGE-M3 captures subtle linguistic nuances and structural modifications that BERT overlooks. Therefore, BGE-M3 was selected as the primary embedding engine, as its contrastive distance provides a reliable and statistically significant basis for identifying complex, cross-lingual plagiarism patterns.

### Classification results

#### Baseline classification results using 500 samples.

The classification outcomes for each model are summarized in Table 3, presenting a comparative analysis of their effectiveness. Model performance was assessed using accuracy, recall, and F1-score across each category.

**Table 3:** The outcomes of seven classifiers for 500 datasets.

	Exact			Semi-Exact			Different		
	Accuracy	Recall	F1 Score	Accuracy	Recall	F1 Score	Accuracy	Recall	F1 Score
**MLP**	0.68	0.62	0.65	0.80	0.80	0.80	0.73	0.80	0.76
**LSTM**	0.68	0.62	0.65	0.80	0.80	0.80	0.73	0.80	0.76
**FNN**	0.57	0.62	0.59	0.83	0.73	0.78	0.76	0.95	0.84
**LR**	0.58	0.52	0.55	0.78	0.78	0.78	0.77	0.85	0.81
**SVM**	0.65	0.52	0.58	0.75	0.80	0.77	0.70	0.70	0.70
**XGBoost**	0.66	0.76	0.65	0.82	0.78	0.80	0.77	0.85	0.81
**RNN**	0.68	0.62	0.65	0.80	0.80	0.80	0.73	0.80	0.76

A more fine-grained evaluation across the three classes provides deeper insight into model behavior and class-specific challenges. The results indicate that most models perform effectively on the Semi-Exact and Different categories, while performance on the Exact class is consistently lower across all methods. In particular, MLP, LSTM, and RNN show similar behavior, achieving good performance in Semi-Exact (up to 80% recall) but lower effectiveness in the Exact category (62% recall, F1 = 0.65). FNN and LR demonstrate comparatively higher discrimination for the Different category, while SVM shows a balanced but overall moderate performance (within the 70–80% range).

Among all models, XGBoost and LSTM achieve the most consistent results, particularly in the Semi-Exact and Different classes, where both recall and F1-scores remain relatively high. However, both models still exhibit weaker performance in the Exact category, highlighting the inherent difficulty of distinguishing highly similar instances in cross-lingual plagiarism detection.

Given the importance of correctly identifying Semi-Exact and Different cases in plagiarism detection tasks, recall and F1-score were considered the primary evaluation metrics, as they better reflect the models’ ability to capture relevant instances while maintaining balanced performance.

According to Table 3, XGBoost and LSTM have demonstrated superior performance in these two metrics. Thus, in the following, their performances are analyzed in detail. The performance metrics and the confusion matrix of the XGBoost classifier are illustrated in Fig 3(a) and Fig 3(b), respectively. A closer analysis of the confusion matrix provides further insights into classification trends:

**Fig 3:**
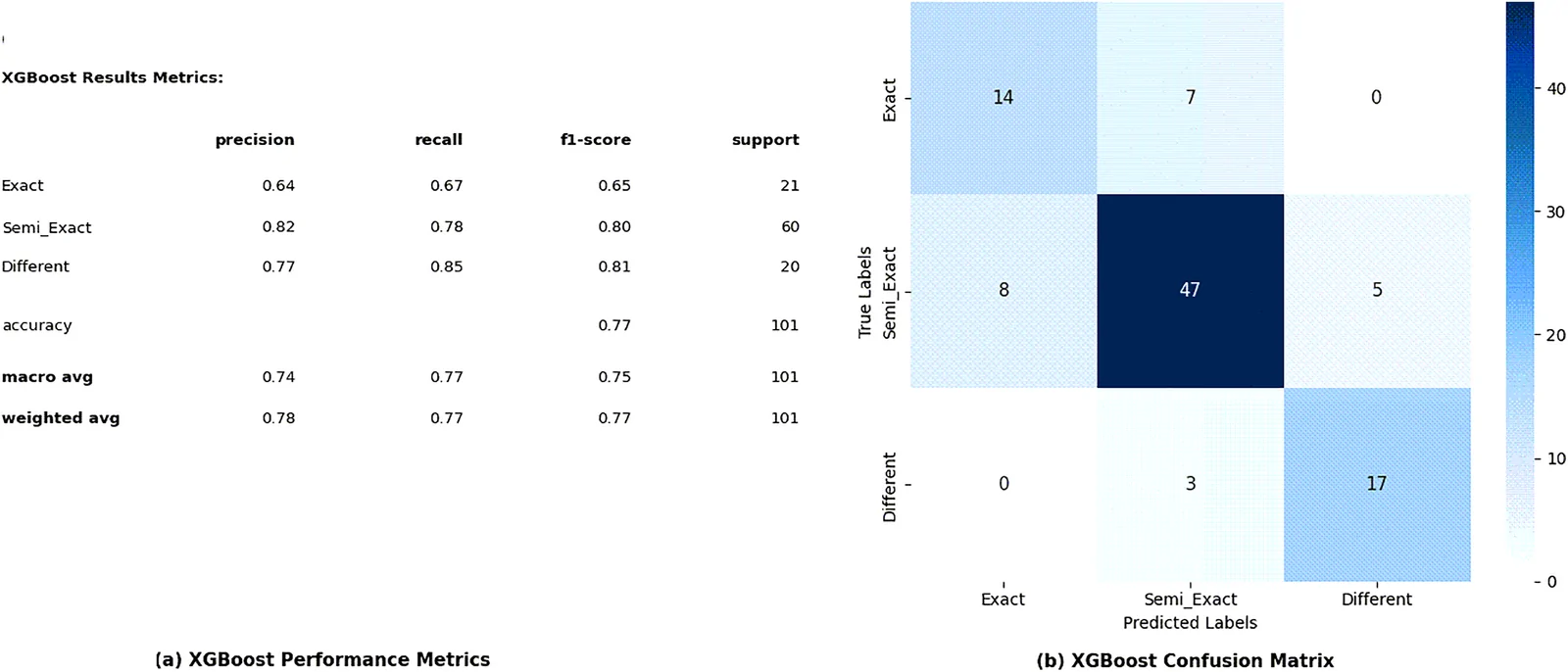
XGBoost (a) performance metrics, (b) Confusion matrix.

The confusion matrix of the XGBoost classifier shows that 14 samples of the Exact class were correctly classified, while 7 were misclassified as Semi-Exact. For the Semi-Exact class, 47 instances were correctly predicted, with 8 misclassified as Exact and 5 as Different. In the Different class, 17 samples were correctly classified, with only 3 misclassified as Semi-Exact.

Overall, most errors occur between neighboring classes (Exact, Semi-Exact and Semi-Exact, Different), indicating that misclassifications mainly arise from semantic overlap rather than random errors.

The performance metrics and the confusion matrix of the LSTM classifier are illustrated in Fig 4. The results show that 13 samples in the Exact category were correctly classified, while 8 were misclassified as Semi-Exact. For the Semi-Exact class, 48 instances were correctly predicted, with 6 misclassified as Exact and 6 as Different. In the Different category, 16 samples were correctly classified, while 4 were assigned to Semi-Exact. Similar to XGBoost, most misclassifications occur between adjacent classes, reflecting semantic overlap and the inherent difficulty of distinguishing borderline plagiarism cases.

**Fig 4:**
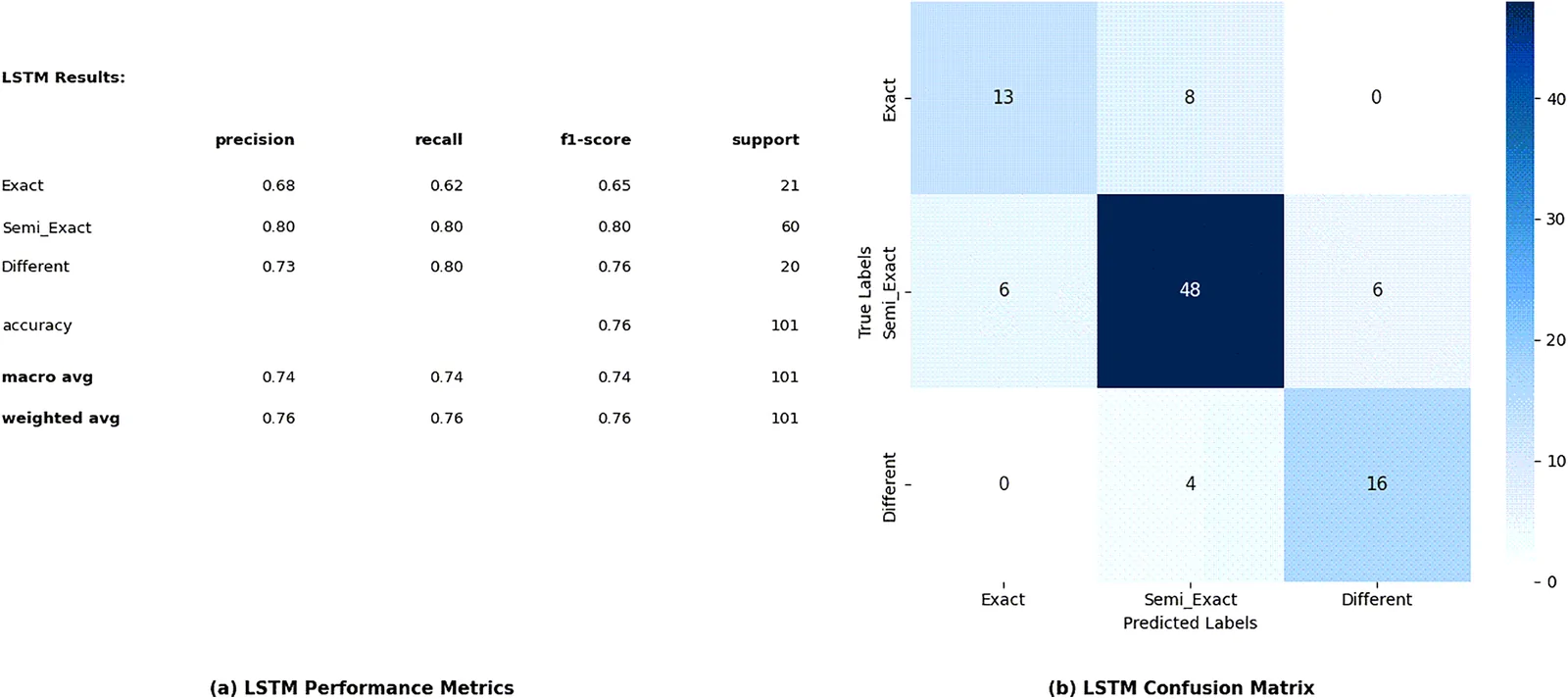
LSTM (a) performance metrics, (b) Confusion matrix.

In both XGBoost and LSTM models, a consistent pattern is observed where the Exact category exhibits lower performance compared to the Semi-Exact and Different classes. This indicates that both models struggle more with identifying strictly exact matches, likely due to high lexical and semantic similarity with the Semi-Exact class, which increases the rate of misclassification.

### Scaled-up experiments results using an expanded dataset

The classification metrics obtained after expanding the dataset to 1,000 samples are presented in Table 4, demonstrate a noticeable improvement in overall model stability and generalization capability compared to the smaller dataset. In particular, increasing the dataset size leads to more reliable discrimination between classes, especially in the Exact and Semi-Exact category, which represents the most ambiguous class in cross-lingual plagiarism detection. A granular examination of the results in Table 4 indicates distinct performance trends across the classification architectures.

**Table 4:** The outcomes of five classifiers for 1000 datasets.

	Exact			Semi-Exact			Different		
	Precision	Recall	F1 Score	Precision	Recall	F1 Score	Precision	Recall	F1 Score
**LSTM**	0.86	0.79	0.82	0.92	0.92	0.92	0.77	0.87	0.82
**LR**	0.90	0.74	0.81	0.92	0.93	0.92	0.75	0.91	0.82
**SVM**	0.90	0.68	0.78	0.90	0.94	0.92	0.77	0.87	0.82
**XGBoost**	0.78	0.84	0.81	0.93	0.90	0.92	0.80	0.87	0.83
**RNN**	0.83	0.66	0.74	0.89	0.93	0.83	0.80	0.87	0.83

The expansion of the dataset to 1,000 samples has significantly stabilized the models, allowing for a clearer differentiation of the discriminative power of each algorithm. Regarding recurrent and sequence models such as LSTM and RNN, these architectures exhibited high stability, particularly in the Semi-Exact category, where LSTM maintained a balanced F1-score of 0.92, thereby confirming the efficacy of gated architectures in capturing semantic dependencies. However, their sensitivity in the Exact class was slightly lower than that of ensemble methods. In comparison, Support Vector Machines (SVM) showed the highest Recall in the Semi-Exact category (0.94), but its performance in the Exact category was the second-lowest (0.68), suggesting a potential for over-generalization in detecting direct translations. Meanwhile, Logistic Regression (LR) demonstrated exceptional robustness in identifying complex plagiarism, notably achieving the highest Recall in the Different category (0.91) and maintaining top-tier Precision (0.90) in the Exact class. Additionally, the XGBoost Classifier proved to be the most effective for detecting near-verbatim plagiarism, achieving the peak Recall of 0.84 in the Exact category, which represents a significant improvement over the previous iteration. Based on the multi-dimensional assessment of these metrics, XGBoost and Logistic Regression were strategically selected for subsequent hyperparameter fine-tuning and optimization.

### XGBoost classifier performance

The performance metrics and the confusion matrix of the XGBoost classifier are illustrated in Fig 5.

**Fig 5:**
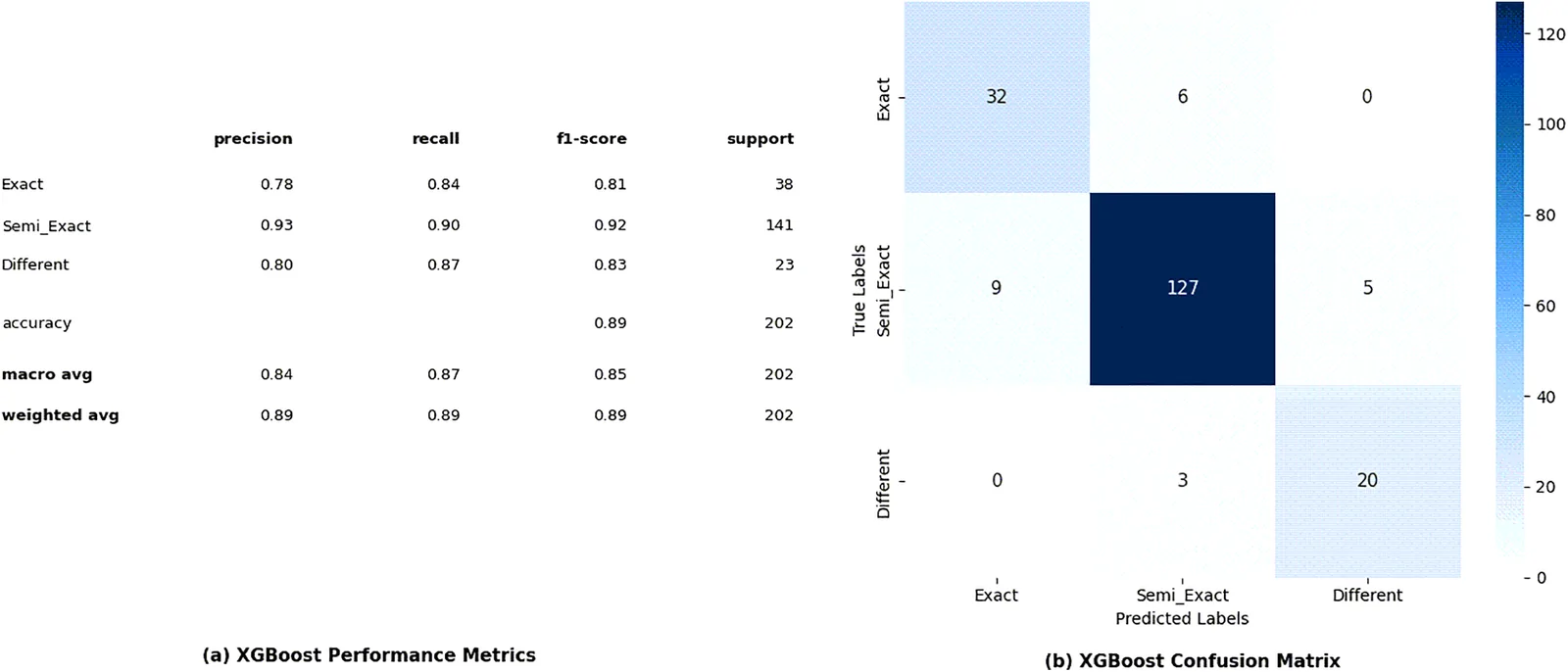
XGBoost (a) performance metrics, (b) Confusion matrix – Scaled up dataset.

The model achieved an overall accuracy of 0.89, demonstrating strong generalization across all categories. Performance was notably superior in the Semi-Exact class (F1: 0.92, Precision: 0.93), proving highly effective in capturing the semantic variations typical of paraphrased content. The Exact and Different classes also showed robust results, with F1-scores of 0.81 and 0.83, respectively, confirming the model’s reliability in distinguishing between varying levels of similarity.

Confusion matrix analysis indicates that misclassifications primarily occur between the Exact and Semi-Exact categories a result of their inherent semantic proximity. Specifically, while some overlap exists between these high-similarity classes, the model maintains a near-perfect distinction between the Exact and Different categories. This clear separation between fully similar and completely dissimilar text pairs validates the model’s discriminative precision in identifying core plagiarism instances while minimizing critical errors.

### Logistic regression performance

The performance of the Logistic Regression model was evaluated using a multiclass confusion matrix and a detailed classification reports are illustrated in Fig 6, respectively.

**Fig 6:**
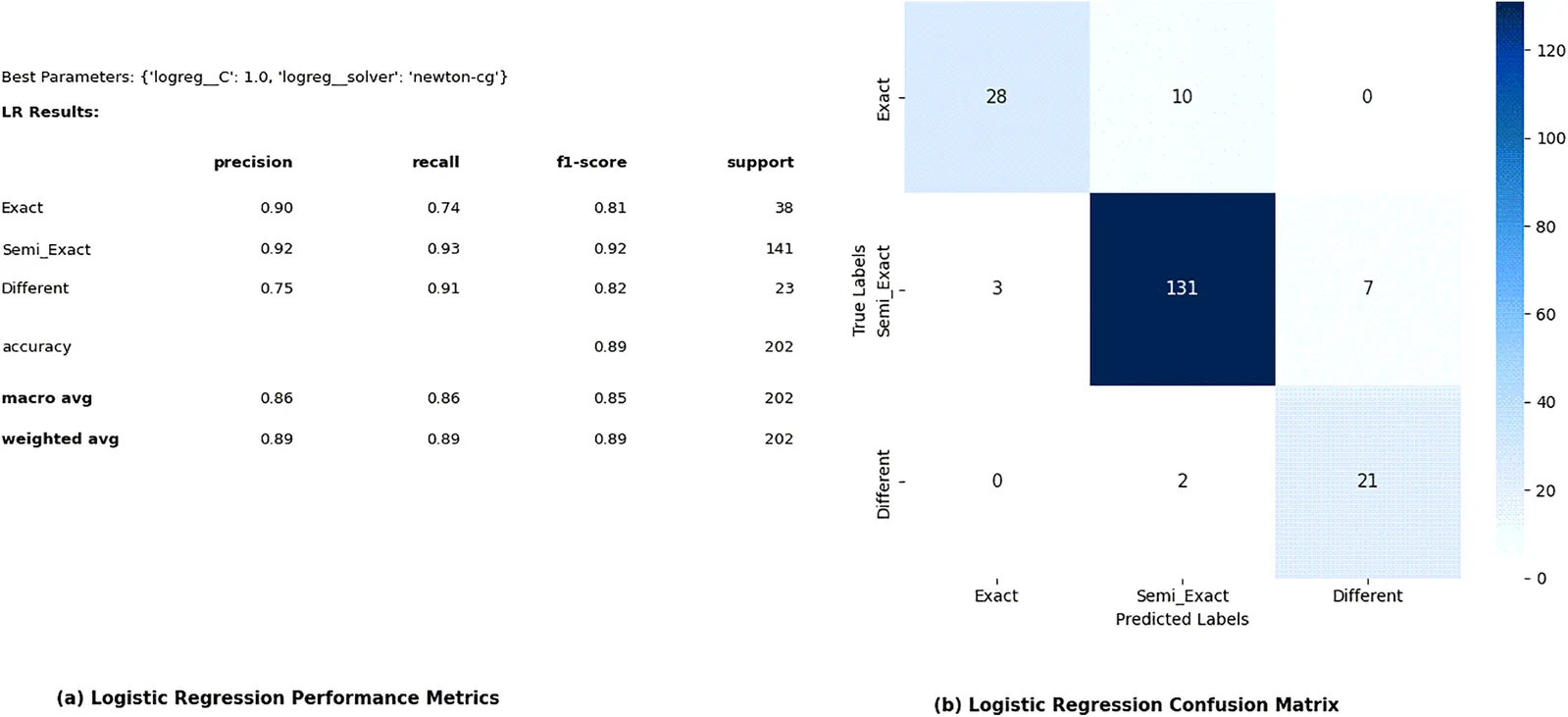
Logistic Regression (a) performance metrics, (b) Confusion matrix- Scaled up dataset.

Logistic Regression model was evaluated using a multiclass confusion matrix and a detailed classification report. The model achieved an overall accuracy of 89%, demonstrating strong predictive capabilities across the three categories: Exact, Semi_Exact, and Different.

As shown in the confusion matrix, the model exhibits exceptional performance in identifying the ‘Semi_Exact’ class, reaching an F1-score of 0.92 and correctly classifying 131 out of 141 instances. The ‘Different’ class also showed high sensitivity (Recall: 0.91), though with a lower precision of 0.75, indicating a slight tendency to misclassify some instances from other categories as ‘Different.’

The most notable challenge for the model was within the ‘Exact’ class, where the recall dropped to 0.74. This suggests that while the model is highly precise when it predicts ‘Exact’ (90%), it fails to capture approximately 26% of the actual ‘Exact’ cases, primarily misclassifying them as ‘Semi_Exact.’ These findings highlight the XGBoost and LR models’ effectiveness in classifying Semi-Exact and Different categories while indicating the need for further refinement to improve performance in the Exact category.

### Fine-tuning strategy

#### Fine tuning for baseline classification.

As illustrated in Table 3, XGBoost and LSTM were selected for fine-tuning due to their complementary strengths. XGBoost was prioritized for its robust handling of class imbalances through gradient boosting, while LSTM was chosen for its ability to capture sequential dependencies and sentence structures, critical for identifying nuanced cross-lingual variations.

#### Fine-Tuning XGBoost for baseline classification.

The comparative results of the XGBoost model before and after the optimization process are detailed in Table 5. Hyperparameter optimization (subsample: 0.4, colsample_bytree: 0.6) improved overall accuracy from 73% to 80%, significantly enhancing generalization.

**Table 5:** Results Before and After Fine-Tuning XGBoost - 500 datasets.

Category	Precision		Recall		F1 Score	
	Before	After	Before	After	Before	After
**Exact**	71%	84%	47%	52%	57%	65%
**Semi-Exact**	76%	80%	83%	85%	79%	83%
**Different**	67%	72%	70%	90%	68%	80%

As shown in Fig 7, the most notable gain occurred in the ‘Different’ category (F1-score: 68% to 80%), while the ‘Semi-Exact’ class remained the most reliable with a Recall of 85%. Despite improvements in the ‘Exact’ category (F1-score: 65%), its Recall (52%) remains a challenge, indicating the difficulty of detecting precise matches compared to paraphrased content.

**Fig 7:**
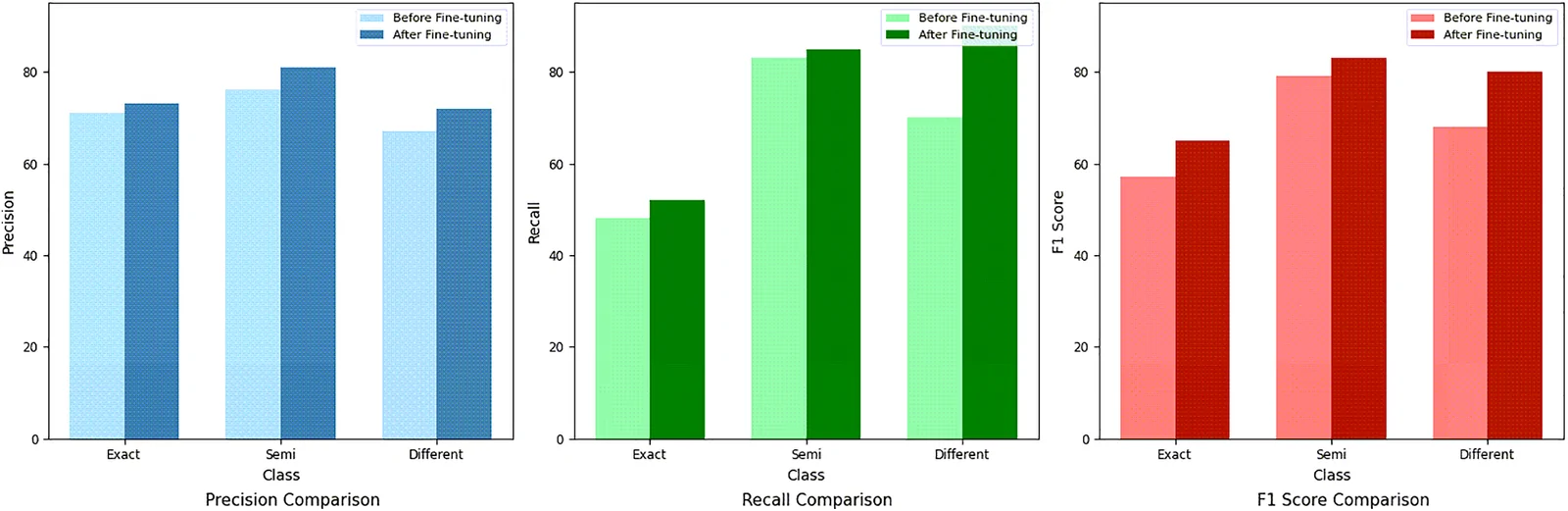
Comparison of Performance Metrics Before and After Fine-Tuning XGBoost- 500 Dataset.

Analysis of confusion matrices in Fig 8. confirms a reduction in misclassifications for non-plagiarized abstracts. However, a semantic overlap remains, with nearly 48% of ‘Exact’ samples misclassified as ‘Semi-Exact.’ This suggests that the boundary between direct translation and heavy paraphrasing remains blurred for tree-based architectures, despite the sampling adjustments which reduced overall false positives.

**Fig 8:**
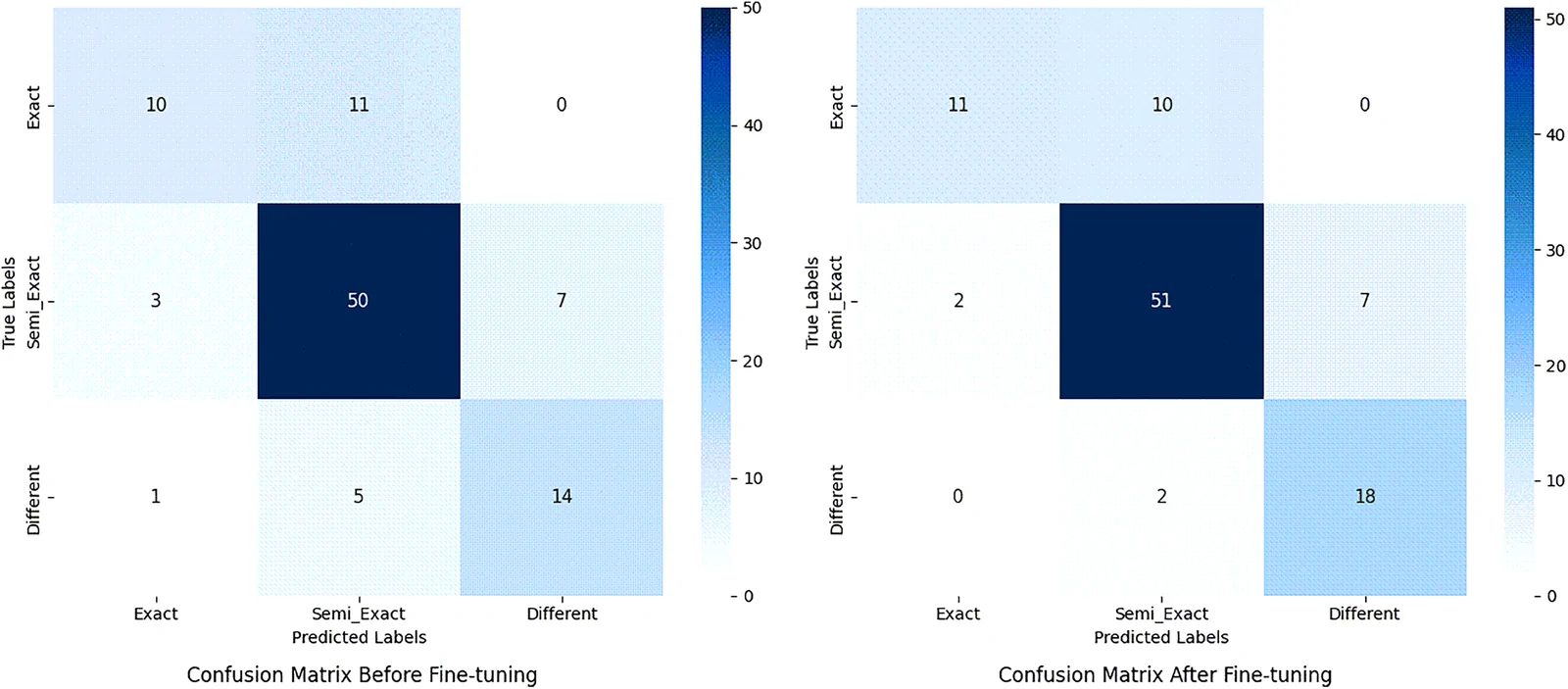
Confusion Matrices Before and After Fine-Tuning XGBoost-500 Dataset.

### Fine-Tuning LSTM for baseline classification

To address the suboptimal performance of the initial recurrent architecture, particularly in the “Exact” and “Different” categories, the LSTM model was systematically optimized. Structural complexity was increased using three layers with 128 hidden units to capture deeper semantic dependencies, while a 0.5 dropout rate and gradient clipping were applied to ensure stability. Furthermore, the learning rate was refined to 0.0005, complemented by early stopping to prevent overfitting. As reported in Table 6, these strategic refinements led to a substantial increase in overall accuracy, rising from 71% to 80%.

**Table 6:** Results Before and After Fine-Tuning the LSTM Model – 500 datasets.

Category	Precision		Recall		F1 Score	
	Before	After	Before	After	Before	After
**Exact**	65%	74%	52%	67%	58%	70%
**Semi-Exact**	74%	82%	80%	83%	77%	83%
**Different**	68%	76%	65%	80%	67%	78%

The impact of this optimization is most evident in the heightened sensitivity toward verbatim plagiarism, where the “Exact” category saw a 15% surge in Recall, raising its F1-score to 70%. Simultaneously, the model’s ability to distinguish independent research the “Different” category improved significantly, with Recall reaching 80%. This enhanced discriminative power, illustrated in the metric charts Fig 9 and confusion matrices Fig 10, confirms that the architectural complexity effectively captures the long-term dependencies required for nuanced paraphrasing.

**Fig 9:**
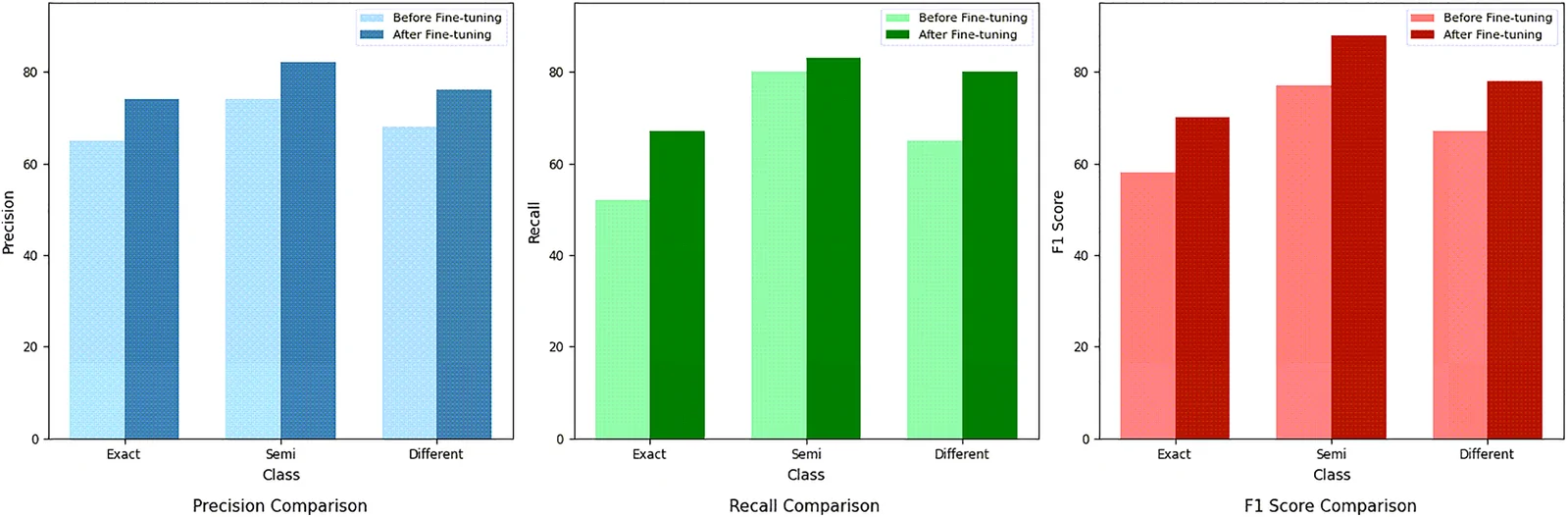
Comparison of Evaluation Metrics Before and After Fine-Tuning the LSTM Model.

**Fig 10:**
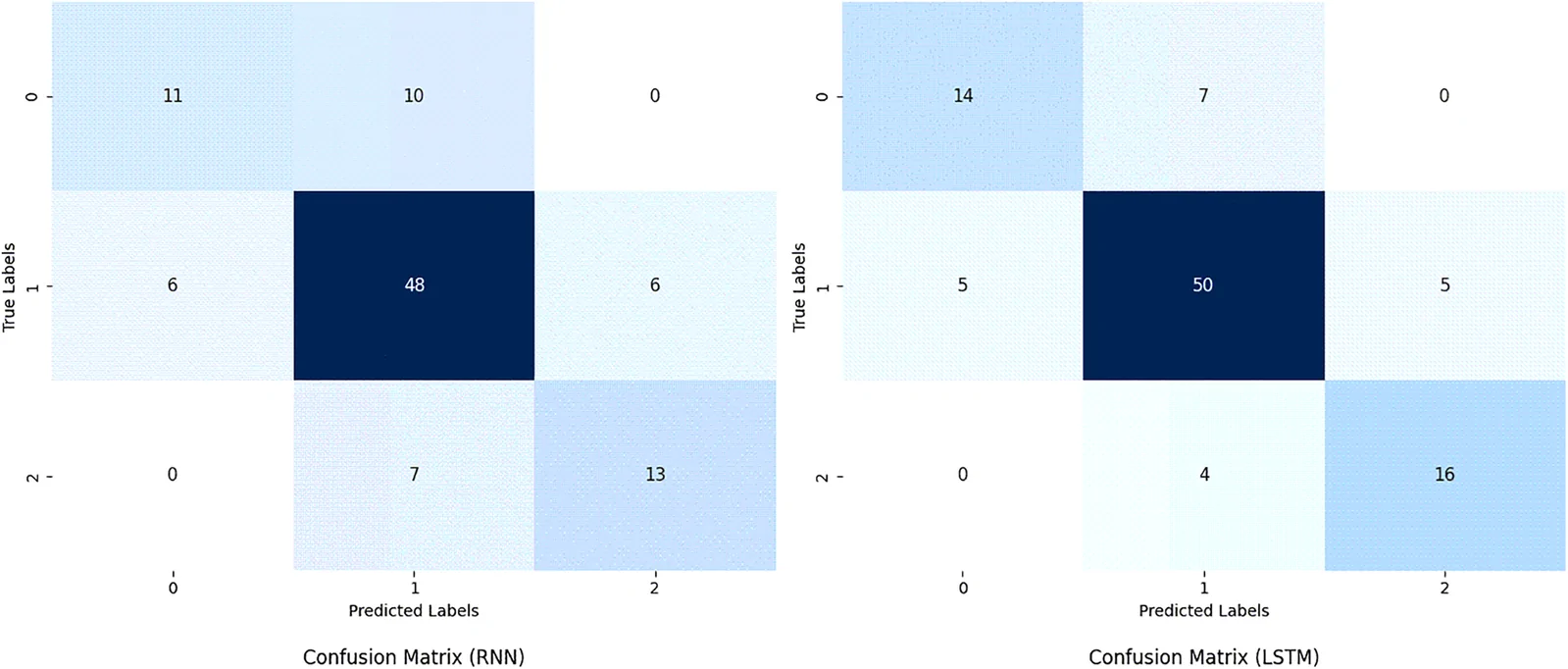
Confusion Matrices Before and After Fine-Tuning the LSTM Model.

However, the “Exact” category remains the most significant challenge. Analysis of the confusion matrices reveals that despite the gains in sensitivity, a portion of “Exact” cases are still misclassified as “Semi-Exact.” This persistent error pattern suggests that the extreme semantic proximity between direct and paraphrased cross-lingual translations creates a boundary that remains partially blurred, even for optimized sequential models. Ultimately, the transition from baseline to optimized LSTM validates that increasing hidden units and stabilizing the learning process is essential for robust pattern recognition in Persian-English embedding spaces.

### Fine tuning for scaled-up classification

Based on the preliminary results in Table 4, Logistic Regression (LR) and XGBoost were selected for further refinement due to their robust and balanced performance across all metrics. While both models achieved comparable overall accuracy, they exhibited distinct strengths: XGBoost proved effective in capturing complex, non-linear feature interactions essential for distinguishing between semantically overlapping classes like “Exact” and “Semi-Exact” whereas, Logistic Regression provided high stability and interpretability, particularly in identifying “Semi-Exact” cases with high precision. Despite these strengths, the initial models showed persistent misclassification patterns in classes with high semantic proximity.

To mitigate these limitations, a systematic hyperparameter tuning process was implemented. This optimization phase aims to enhance model sensitivity to subtle cross-lingual semantic variations, improve class separation between highly similar plagiarism categories, and strengthen generalization across different levels of text similarity within the 1,000-sample dataset.

### Fine-Tuning XGBoost for scaled-up classification

Experimental results demonstrate a strong correlation between dataset scale and predictive accuracy. Expanding the dataset to 1,000 samples enabled the XGBoost model to better capture intricate cross-lingual patterns while mitigating overfitting and enhancing generalization. Following this expansion, automated hyperparameter tuning via the Optuna framework yielded marginal improvements, as summarized in Table 7, with accuracy rising from 0.89 to 0.90 and the macro F1-score from 0.85 to 0.87. This minimal gain suggests that the model has reached an architectural saturation point. At this scale, the tree-based architecture has effectively exhausted the learning capacity of the current feature set. This indicates that further performance breakthroughs are likely constrained by the inherent semantic complexity of Persian-English plagiarism rather than algorithmic configuration, marking a plateau in the model's optimization potential.

**Table 7:** Results Before and After Fine-Tuning XGBoost - 1000 datasets.

Category	Precision		Recall		F1 Score	
	Before	After	Before	After	Before	After
**Exact**	0.78	0.77	0.84	0.89	0.81	0.83
**Semi-Exact**	0.93	0.95	0.90	0.89	0.92	0.92
**Different**	0.80	0.81	0.87	0.91	0.83	0.86

Analysis of the confusion matrices in Fig 11, reveals several key insights, that the model effectively discriminates non-plagiarized content, maintaining a near-zero false-positive rate for the ‘Different’ class. A strategic trade-off was observed as Recall for the ‘Exact’ and ‘Different’ categories increased to 0.89 and 0.91, respectively. While this led to a minor reduction in Precision, the gain in Recall is prioritized in plagiarism detection to minimize critical false negatives.

**Fig 11:**
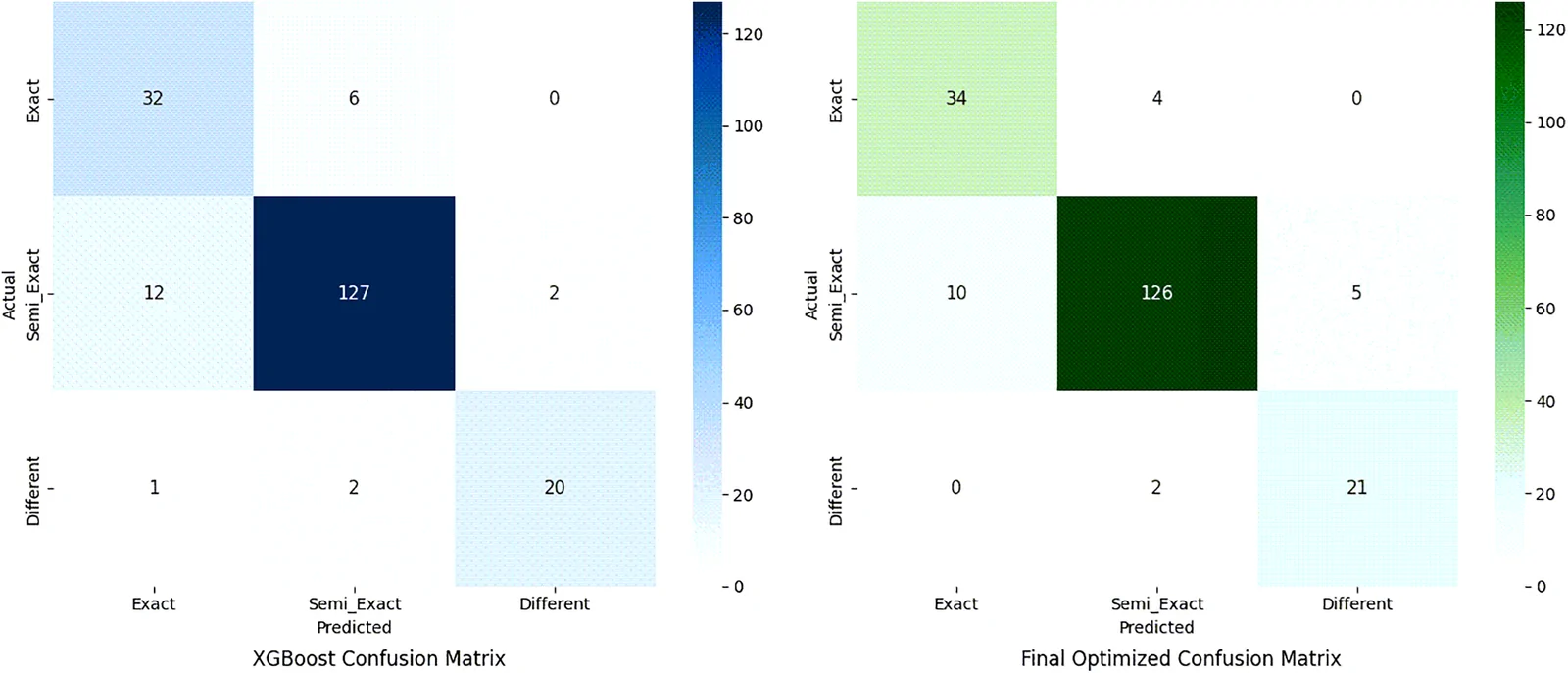
Confusion Matrices Before and After Fine-Tuning XGBoost-1000 Dataset.

As illustrated in the performance charts in Fig 12, the persistent overlap between ‘Exact’ and ‘Semi**-**Exact’ samples highlights a performance ceiling for gradient-boosted decision trees; the blurred boundary between direct translation and heavy paraphrasing remains a significant semantic challenge.

**Fig 12:**
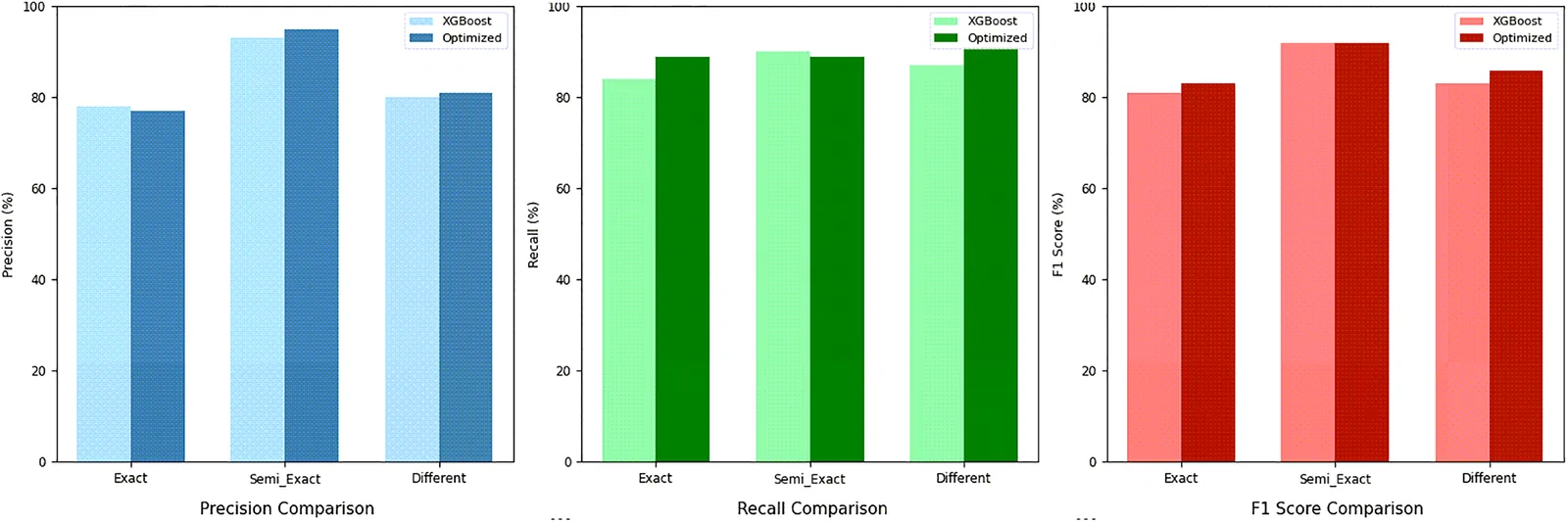
Comparison of Performance Metrics Before and After Fine-Tuning XGBoost- 1000 Dataset.

Despite this, as shown in the performance charts, F1-scores consistently remain above 80%, with high Precision in the ‘Semi-Exact’ category ensuring a robust system that minimizes the risk of false accusations in academic integrity contexts.

### Fine-tuning logistic regression for scaled-up classification

Following the initial experiments, Logistic Regression was further optimized, demonstrating a substantial performance leap across all metrics. As detailed in Table 8, the F1-score for the “Exact” class soared from 0.81 to 0.96, while the “Semi-Exact” category reached an exceptional F1-score of 0.97.

**Table 8:** Results Before and After Fine-Tuning LR - 1000 datasets.

Category	Precision		Recall		F1 Score	
	Before	After	Before	After	Before	After
**Exact**	0.90	0.95	0.74	0.97	0.81	0.96
**Semi-Exact**	0.92	0.98	0.93	0.96	0.92	0.97
**Different**	0.75	0.84	0.91	0.91	0.82	0.88

The superior performance of Logistic Regression in this task can be attributed to the nature of the high-dimensional embedding space. The dense semantic representations generated by the language model provide a highly structured feature space where class boundaries tend toward linear separability. In such high-dimensional environments, a discriminative linear classifier like Logistic Regression is often more effective than complex non-linear models. It effectively minimizes the risk of overfitting (low variance) while precisely modeling the linear decision boundaries between different plagiarism types.

The transition shown in the confusion matrices in Fig 13 illustrates a near perfect classification for the “Exact” plagiarism class, with only one misclassification.

**Fig 13:**
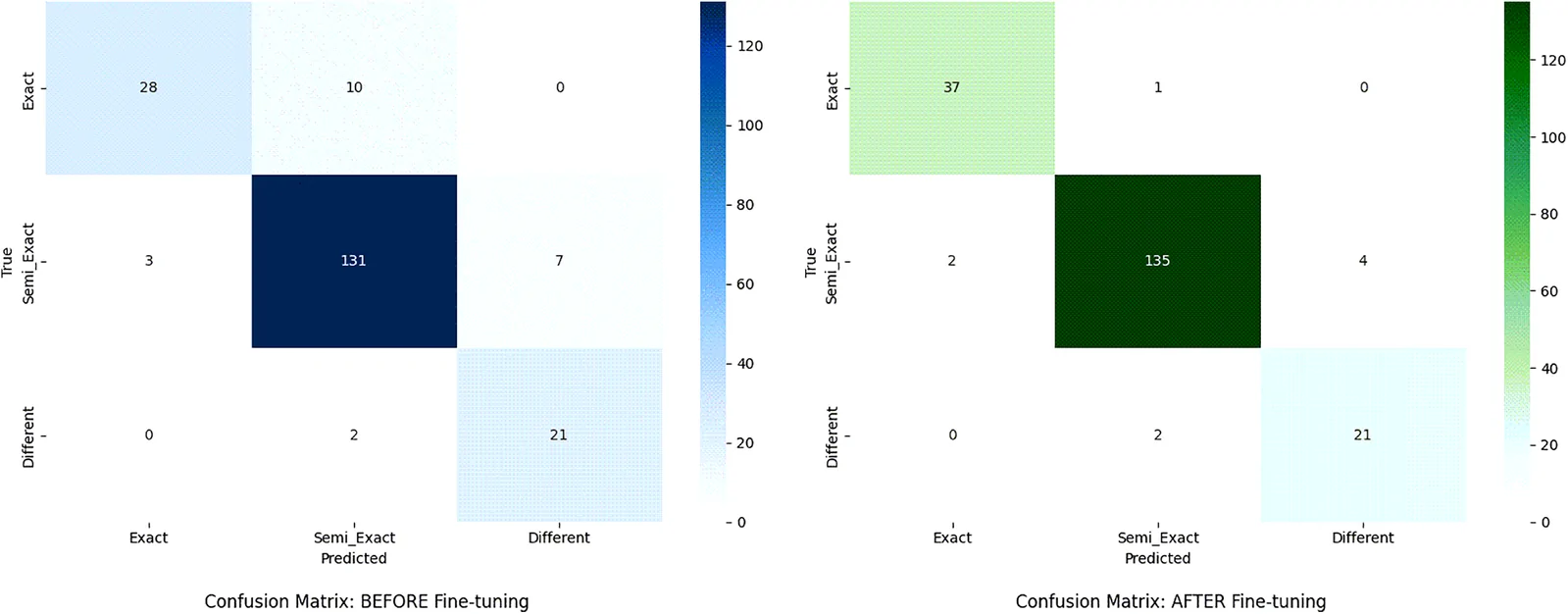
Comparison of Performance Metrics Before and After Fine-Tuning LR- 1000 Dataset.

The fine-tuning results in Fig 14., process significantly improved the model's Recall for the “Exact” class (from 0.74 to 0.97), indicating that the model became highly sensitive to true plagiarism instances. This gain was achieved without a “Precision-Recall trade-off,” as Precision also improved to 0.95, confirming that the model's confidence in its predictions is rooted in robust semantic patterns rather than random noise.

**Fig 14:**
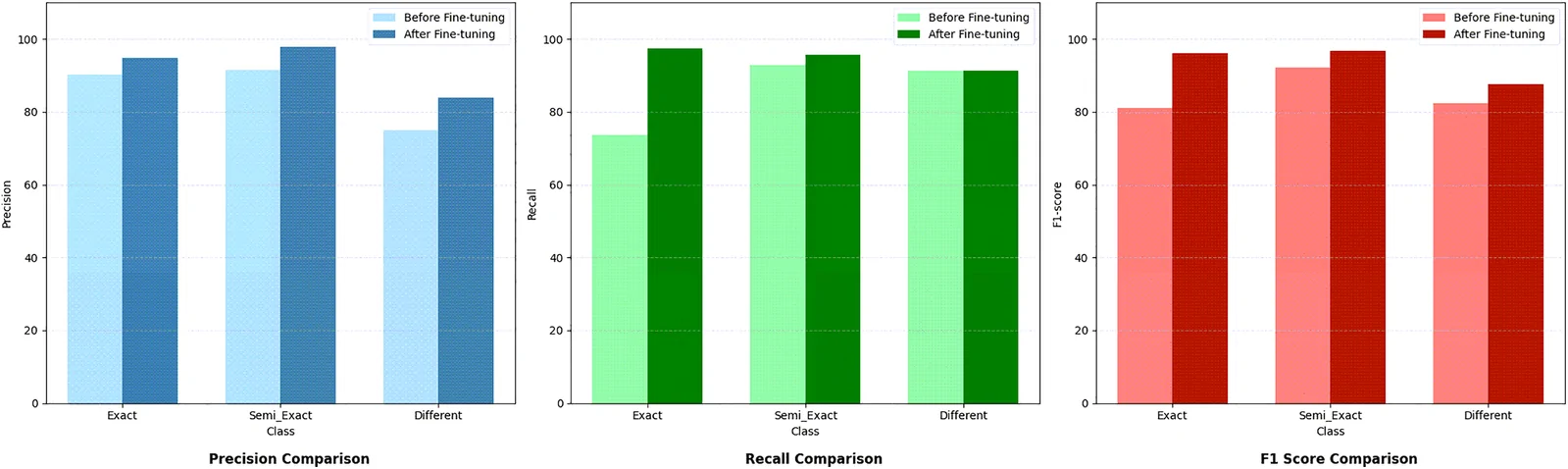
Comparison of Performance Metrics Before and After Fine-Tuning LR- 1000 Dataset.

### Statistical significance testing

To ensure that the observed performance gains were not a result of stochastic variation or random chance, a McNemar’s Test for statistical significance was performed. This test is specifically suited for comparing the performance of two classifiers on the same dataset. As shown in Fig 15, the test yielded a p-value of 0.00046.

**Fig 15:**
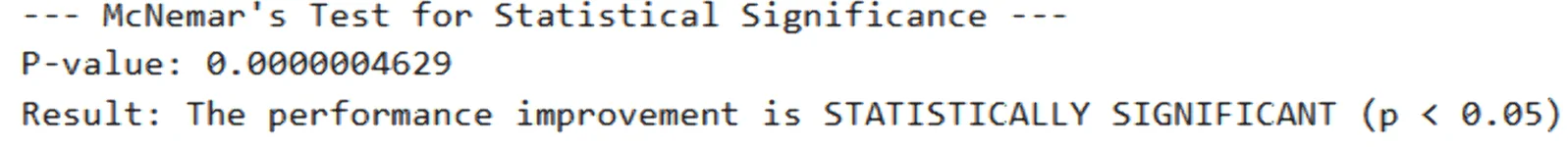
McNemar’s Test for statistical significance.

This extremely low P-value provides strong empirical evidence that the improvement from the baseline to the fine-tuned Logistic Regression model is statistically significant. This result reinforces the reliability of the proposed system, confirming that the optimization process led to a genuine enhancement in the model's ability to detect cross-lingual plagiarism between Persian and English.

## Discussion

From an algorithmic perspective, the superior performance of Logistic Regression (LR) over non-linear models like XGBoost and LSTM suggests that the semantic embedding space generated by BGE-M3 may exhibit a relatively high degree of linear separability for this task. The BGE-M3 model projects text into a dense, 1024-dimensional semantic space, where semantically similar documents are positioned closer together. Within this embedding space, LR appears to construct an effective decision boundary that generalizes well while maintaining low model complexity. In contrast, XGBoost may derive less benefit from recursive feature partitioning in dense continuous embedding spaces, leading to limited performance gains over simpler linear classifiers. Similarly, because BGE-M3 compresses contextual and sequential information into a single fixed-length semantic representation, the sequential modeling capability of LSTM may provide limited additional discriminative information. These findings suggest that, for low-resource cross-lingual plagiarism detection, the quality of semantic representations may play a more important role than increasing downstream classifier complexity.

Linguistically, these findings suggest that the embedding model effectively mitigates syntactic disparities between Persian (Subject–Object–Verb) and English (Subject–Verb–Object). While conventional classifiers often struggle to bridge these cross-lingual differences with limited training data, BGE-M3 appears to map semantically equivalent concepts into a shared representation space during pre-training, regardless of surface-level linguistic structure. As a result, downstream classification becomes largely dependent on semantic proximity within the embedding space, enabling LR to effectively discriminate between plagiarism categories without relying on complex non-linear feature interactions.

Furthermore, expanding the dataset from 500 to 1,000 samples improved model stability and reduced ambiguity between the Exact and Semi-Exact plagiarism classes. However, the persistent confusion between these categories underscores the ongoing technical challenge of distinguishing literal translations from highly faithful paraphrases. These findings are consistent with previous studies highlighting the advantages of semantic embedding models over conventional translation-then-monolingual pipelines. Moreover, the results suggest that carefully optimized conventional machine learning classifiers can achieve performance comparable to more complex deep learning architectures when supported by high-quality multilingual semantic representations, while offering lower computational complexity and greater interpretability.

## Conclusions

This study investigated cross-lingual plagiarism detection in Persian–English academic abstracts, specifically addressing the challenges posed by machine translation and complex paraphrasing. By leveraging the BGE-M3 embedding model and expanding the dataset to 1,000 samples, the empirical results demonstrated that downstream classifier selection becomes increasingly important as dataset size increases. A key finding of this research is the superior generalization performance of the optimized Logistic Regression (LR) model on the expanded dataset. While XGBoost and LSTM showed strong performance on the initial 500-sample dataset, the optimized LR model achieved the best overall performance on the larger dataset. Ultimately, the proposed framework provides a computationally efficient and scalable solution for cross-lingual plagiarism detection in academic publishing systems.

However, the consistent difficulty in identifying the Exact plagiarism category across all models, attributed to the extreme semantic proximity between verbatim and near-verbatim translations, remains a key technical challenge that warrants further methodological investigation.

To further enhance the effectiveness and reliability of cross-lingual plagiarism detection, future research should explore the integration of syntactic information, Named Entity Recognition (NER), and authorship-related metadata to improve plagiarism detection performance. Developing Explainable AI (XAI) techniques could provide transparent justifications for plagiarism decisions, increase user trust, and reduce the impact of false-positive detections. From a practical perspective, applying model pruning and quantization may improve computational efficiency and facilitate real-time, large-scale deployment in academic publishing workflows. In addition, incorporating continual learning mechanisms would enable plagiarism detection systems to adapt to evolving language patterns and emerging AI-assisted plagiarism strategies

Finally, future studies should investigate class imbalance mitigation techniques, including oversampling and class-weighted loss functions, to improve model reliability in real-world environments where genuine documents substantially outnumber plagiarized ones. Expanding the proposed framework to larger and more diverse datasets spanning multiple scientific disciplines and additional cross-lingual settings, particularly low-resource language pairs, would further improve the generalizability, robustness, and practical applicability of the proposed methodology.
