# Supplementary material for: English-Persian cross-language plagiarism detection: Multilingual deep learning approach
Source: PLoS One. 2026 Sep 30;21(9):e0354459. doi: 10.1371/journal.pone.0354459 (PMC13626316; doi:10.1371/journal.pone.0354459)
Supplement: Supplementary file 1 [file pone.0354459.s001.pdf]

Paula Beatty's Comments to Authors:

Overall, this research manuscript offers novel insight as to how machine learning can address a gap in cross-cultural communication and plagiarism detection. While the background information section is detailed and helps contextualize the research, perhaps clarifying why XGBoost and LSTM are chosen when the other models seem to perform just as strong if not more so.

Major Comments:

1. What is the protocol for interpreting recall, accuracy, and F1 scores? What threshold is considered a strong or weak score? In the analysis section, "moderate" is a term to describe SVM's performance of 80% recall, whereas XGBoost is considered "strong" for 78% in the next paragraph. Clarifying how these metrics are intended to be interpreted might be helpful for the reader in understanding the results.
2. A possible limitation is that the data is not readily available. Readers must go to a GitHub repository to access the data for this research. Ensuring that this data is more easily accessible could help with reproducibility.
3. Adding specific studies and references to section "2.4 Research gap" will strengthen the section and tie together the thorough background section.
4. While the fine tuning of the hyperparameters is clearly outlined in the methods section, it might be helpful to add a more thorough statistical analyses, such as p-values, to help the reader quantify the benefit of these adjustments.

References

1. Ansar, S, Zarinbal, M, Mohebi, A. English-Persian cross-language plagiarism detection: Multilingual deep learning approach. Public Library Of Science One. Forthcoming 2026.
