# Supplementary material for: English-Persian cross-language plagiarism detection: Multilingual deep learning approach
Source: PLoS One. 2026 Sep 30;21(9):e0354459. doi: 10.1371/journal.pone.0354459 (PMC13626316; doi:10.1371/journal.pone.0354459)
Supplement: Supplementary file 2 [file pone.0354459.s003.docx]

**Manuscript Title:** *English-Persian cross-language plagiarism detection: Multilingual deep learning approach*

**Journal:** *Information Discovery and Delivery*
**Manuscript ID:** *PONE-D-25-53532*

**Dear Dr. Bolboacă,**

Thank you for your email and for the opportunity to submit a revised version of our manuscript. We appreciate the constructive feedback provided by you and the reviewers, which has helped us significantly improve the quality and robustness of our study.

We have addressed all the comments raised by the reviewers and the editorial requirements. Most notably, we have:

- Expanded our dataset from 501 to 1,000 samples to ensure better generalizability.
- Included statistical significance testing (McNemar’s test) to validate the model comparisons.
- Made our dataset and source code publicly available on GitHub to comply with PLOS ONE’s data availability policy.
- Refined the manuscript's structure for better conciseness, particularly in the Introduction and Conclusion sections.

Below, we provide a point-by-point response to the editor’s and reviewer’s comments.

***Response to Journal Requirements:***

- Style Requirements: We have reviewed the PLOS ONE style templates and ensured that the manuscript formatting and file naming comply with the journal’s standards.
- Code and Data Sharing: In accordance with the journal's policy, we have made our author-generated code and the anonymized dataset publicly available. The repository can be accessed at: *https://github.com/SaRaABlue/English-Persian-cross-language-plagiarism-detection-multilingual-deep-learning-approach.git*
- Data Availability Statement: We have updated our statement to reflect that all data underlying the findings are now available in a public repository without restrictions.

***Response to Reviewer #1***

**Reviewer Comment: “The theme of the article English-Persian cross-language plagiarism detection using a multilingual deep learning approach, sound and is relevant and timely... The focus on Persian-English plagiarism detection fills a notable research gap.”**

**Response:**

We sincerely thank the reviewer for the positive and encouraging feedback regarding the importance and novelty of our work. We are pleased that the relevance of focusing on the English-Persian language pair was recognized.

**1. Structure and Clarity**

**Reviewer Comment: “There are instances where the language could be more concise... For example, the introduction and conclusion sections seem very detailed... Some technical terms, such as "contrastive distance," are introduced without sufficient explanation.”**

**Response:**

We appreciate this constructive suggestion. We have revised the Introduction and Conclusion sections to be more concise and focused on the core contributions and findings. Furthermore, we have added a clear definition and technical explanation of **"contrastive distance"** in the Methodology section (Page 16, Line 284) to ensure better understanding for all readers.

**1. Introduction - Page 3 – Line 36**

The proliferation of sophisticated machine translation tools has significantly escalated the risk of cross-lingual plagiarism, a form of academic misconduct where content is translated and presented as original work without proper attribution [1]. Unlike monolingual plagiarism, cross-lingual detection requires capturing deep semantic invariants across disparate linguistic structures, rendering traditional keyword matching techniques ineffective. This challenge is particularly acute for the Persian–English language pair, where detection systems are hindered by a critical scarcity of benchmark datasets and the complex morphological nuances of the Persian language.

To bridge this gap, this paper introduces a robust deep learning (DL) framework specifically engineered for Persian–English cross-lingual detection. The primary contributions of this work include the development of a systematic, AI-augmented dataset designed to reflect high contextual fidelity and modern translation patterns and the implementation of a multi-stage classification pipeline. By optimizing the synergy between high dimensional embedding models and fine-tuned classifiers, the proposed methodology not only improves detection accuracy but also enhances the model's ability to generalize across varying levels of semantic paraphrasing.

The paper is structured as follows: Section 2 critically reviews existing literature and identifies the research gap; Section 3 details the proposed methodology and architectural design; Section 4 presents the experimental evaluation and statistical validation; and Section 5 concludes the study with a discussion on broader implications and future research directions.

**Contrastive Distance Page 14 – Line 243**

Contrastive Distance in the context of Large Language Models (LLMs) and representation learning is a metric used to evaluate the proximity of semantic embeddings within a high-dimensional vector space. It operates on the principle of distance-based discrimination, where the model is tasked with minimizing the distance between 'positive' pairs (e.g., a Persian abstract and its accurate English translation) while simultaneously maximizing the gap for 'negative' or dissimilar pairs (e.g., unrelated or heavily manipulated texts) [37] .

**5. Conclusion - Page 34- Line 555**

This study investigated cross-lingual plagiarism detection in Persian–English academic abstracts, specifically addressing the challenges posed by machine translation and complex paraphrasing. By leveraging the BGE-M3 embedding model and expanding the dataset to 1,000 samples, we demonstrated that as data scales, the choice of classifier becomes a critical factor in maintaining robustness. The core finding of this research is the superior performance of Logistic Regression (LR) over more complex architectures like XGBoost and LSTM when applied to high-dimensional embedding spaces in a larger dataset. While XGBoost and LSTM showed strong performance on the initial 500-sample set, the optimized LR model achieved the best overall performance and generalization on the expanded data. The results indicate that semantic representations from BGE-M3 create a near-linearly separable structure, where simpler linear models provide higher stability (P< 0.05) while complex models may reach early performance saturation. The proposed framework offers a computationally efficient solution for academic publishing systems.

However, the consistent difficulty in identifying the "Exact" plagiarism category across all models attributed to the extreme semantic proximity between verbatim and near-verbatim translations remains a key technical challenge.

Future Research Directions To further enhance the effectiveness and reliability of cross-lingual plagiarism detection, the following areas are proposed for future investigation:

- Linguistic & Metadata Integration: Incorporating syntactic structures, Named Entity Recognition (NER), and metadata (e.g., authorship patterns) to improve detection accuracy.
- Interpretability & Ethics: Developing Explainable AI (XAI) methods to provide transparent justifications for flagged content and mitigate the impact of false positives.
- Computational Efficiency: Applying model pruning and quantization to facilitate real-time, large-scale deployment in publishing workflows.
- Continuous Learning: Implementing adaptive mechanisms that allow models to learn from evolving language patterns and emerging plagiarism techniques.
- Cross-Disciplinary Expansion: Validating the framework on larger, more diverse datasets across various scientific disciplines to ensure global generalizability.
- Imbalance Handling: Investigating techniques such as oversampling or class-weighted loss functions to ensure model reliability in real-world settings where non-plagiarized cases significantly outnumber instances of misconduct.
- Cross-Lingual & Disciplinary Expansion: Expanding and validating the framework on a broader range of language pairs (particularly low-resource languages) and diverse scientific disciplines to ensure the global generalizability and robustness of the proposed methodology.

**2. Dataset Size and Generalizability**

**Reviewer Comment: “The dataset consists of only 501 abstracts, which is relatively small... This could lead to biased or overfitted results.”**

**Response:**

We completely agree that a larger dataset enhances the reliability of the model. As suggested, we have expanded the dataset from 501 to 1,000 samples. This larger and more diverse dataset has been used to re-train and re-evaluate all models. The updated results in Section 4 confirm that the models’ maintain high performance while offering better generalizability.

**4.1. Dataset - Page 10 – Line 164**

The empirical foundation of this study comprises a curated dataset of 1,000 academic abstracts sourced from the Iranian Research Institute for Information Science and Technology (IranDoc). To facilitate the investigation of cross-lingual plagiarism detection between Persian and English, the original Persian abstracts underwent a systematic translation and modification pipeline. While traditional methodologies have frequently utilized conventional machine translation (MT) engines, this research intentionally employed Microsoft Copilot for the translation phase. This selection was motivated by two primary factors: first, the advanced capability of large language models (LLMs) to preserve nuanced semantic structures in technical discourse [32]; and second, the necessity for the detection model to remain robust against modern, AI-mediated translation artifacts that researchers may encounter in contemporary academic environments.

**3. Dataset Distribution (Balance vs. Imbalance)**

**Reviewer Comment: “The dataset is evenly distributed across three categories (Exact, Semi-Exact, Different), which may not reflect real-world scenarios where plagiarism cases are often imbalanced. This could affect the model's performance in practical applications.”**

**Response:**

We sincerely thank the reviewer for this insightful and valuable observation. We agree that real-world plagiarism scenarios are typically imbalanced. In this study, we deliberately maintained a balanced dataset to establish a clear performance baseline and prevent the models from developing a bias toward the majority class during the initial evaluation phase.

However, we fully recognize the importance of this point for practical applications. Following the reviewer's excellent suggestion, we have updated our Future Work section to emphasize the need for evaluating model robustness in imbalanced settings using techniques such as oversampling or class-weighted loss functions. We believe this addition provides a more comprehensive perspective for future researchers.

**Page 36 – Line 583**

- Imbalance Handling: Investigating techniques such as oversampling or class-weighted loss functions to ensure model reliability in real-world settings where non-plagiarized cases significantly outnumber instances of misconduct.

**4. Model Performance (BGE-M3 vs. BERT)**

**Reviewer Comment: “The study does not explore why BERT underperformed or whether additional fine-tuning could improve its results.”**

**Response:**

We sincerely thank the reviewer for this technical suggestion. We have significantly expanded our comparative analysis in Section 4.2 and added Figure 2 to visually demonstrate why BGE-M3 outperforms BERT in this specific task.

Our analysis reveals that BERT suffers from "representation collapse" in this context; as shown in Fig 2a, it generates a narrow distribution of similarity scores that fails to provide a clear decision boundary between categories. In contrast, BGE-M3 utilizes a contrastive learning architecture specifically optimized for multilingual alignment. This allows it to yield a stratified distribution of scores across a wide dynamic range (24% to 92%), effectively isolating "Exact," "Semi-Exact," and "Different" translations into distinct clusters.

By capturing these subtle linguistic nuances that BERT overlooks, BGE-M3 provides a much more reliable foundation for cross-lingual plagiarism detection. We have also clarified in the manuscript that while fine-tuning was considered, the inherent architectural advantages of BGE-M3’s contrastive distance make it the superior choice for this application.

**Section 4.2 – Page 14 – Line 249:**

The comparative analysis of embedding models, as visualized in Fig 2, demonstrates a significant disparity in their capacity to represent cross-lingual semantic relationships. The BERT embeddings (Fig.2a) , exhibit high intra-cluster density with negligible variance between categories. This indicates that BERT suffers from representation collapse in this context; it generates a narrow distribution of similarity scores that fails to distinguish between an exact translation and a sophisticatedly paraphrased text. Consequently, the contrastive distance in the BERT space is insufficient to provide a clear decision boundary for plagiarism detection.

In contrast, the BGE-M3-Embeddings (Fig. 2b) yield a robust and stratified distribution of similarity scores, spanning a wide dynamic range (approximately 24% to 92%). The effectiveness of BGE-M3 is attributed to its contrastive learning architecture, which is specifically optimized to maximize the contrastive distance between semantically distinct pairs.

As illustrated, BGE-M3 successfully isolates the three translation categories (Exact, Semi-Exact, and Different) into observable clusters. This high discriminative resolution proves that BGE-M3 captures subtle linguistic nuances and structural modifications that BERT overlooks. Therefore, BGE-M3 was selected as the primary embedding engine, as its contrastive distance provides a reliable and statistically significant basis for identifying complex, cross-lingual plagiarism patterns.

While BERT has substantial computational overhead, the evaluations highlighted the effectiveness of BGE-M3-Embeddings for this application. By combining advanced cross-lingual alignment capabilities with reduced operational complexity, BGE-M3-Embeddings emerged as the optimal solution for addressing this study's requirements. Thus, BGE-M3-Embeddings is selected as the foundational representation framework for implementing cross-lingual plagiarism detection, emphasizing performance and scalability in resource-constrained settings.

Fig 2. Contrastive distance of (a) BERT, (b) BGE-M3

Fig 3. Contrastive distance of (a) BERT, (b) BGE-M3

|  |  |
| --- | --- |
| (a) | (b) |

**5. Performance in the "Exact" Category**

**Reviewer Comment: “Both XGBoost and LSTM models struggled with the Exact category, showing lower recall and F1 scores... indicates that the models may not be fully optimized for detecting direct translations or detecting exact plagiarism.”**

**Response:**

We appreciate the reviewer for highlighting this critical point. We have addressed this concern by expanding the dataset to 1,000 samples, which provided the models with a more comprehensive and diverse set of training examples.

With this increased data volume, we re-evaluated our classification framework and observed a significant performance boost across all categories. In particular, the Logistic Regression model, when paired with our refined embedding space, demonstrated exceptional robustness in the "Exact" category. The increased data allowed the models to better learn the subtle decision boundaries between exact translations and sophisticated paraphrasing.

As presented in the updated Section 4.5.2 Fine Tuning for Scaled-Up Classification and summarized in Table 8, the recall and F1-scores for the "Exact" category have improved substantially, reaching 97% and 96% .This proves that the initial fragility was primarily due to the limited sample size rather than a structural flaw in the methodology. We have updated the manuscript to reflect these improved metrics and added a discussion on how the expanded dataset mitigated the previous limitations.

**Section 4.5.2 Fine Tuning for Scaled-Up Classification Page 32 – Line 530**

**Table 8- Results Before and After Fine-Tuning LR - 1000 datasets**

| **Category** | **Precision** | | **Recall** | | **F1 Score** | |
| --- | --- | --- | --- | --- | --- | --- |
|  | **Before** | **After** | **Before** | **After** | **Before** | **After** |
| **Exact** | 0.90 | 0.95 | 0.74 | 0.97 | 0.81 | 0.96 |
| **Semi-Exact** | 0.92 | 0.98 | 0.93 | 0.96 | 0.92 | 0.97 |
| **Different** | 0.75 | 0.84 | 0.91 | 0.91 | 0.82 | 0.88 |

**6. Dependency on Translation Tools (Microsoft Copilot)**

**Reviewer Comment: “The study relies heavily on Microsoft Copilot for translation, which may introduce biases or errors inherent in the tool. The accuracy of the plagiarism detection system is contingent on the quality of the translations...”**

**Response:**

We sincerely thank the reviewer for this insightful comment. We would like to clarify that the decision to employ Microsoft Copilot for the translation phase was a deliberate methodological choice rather than a limitation.

As detailed in the revised Section 4.1 Dataset, this selection was motivated by two primary factors:

- Semantic Fidelity: The advanced capabilities of Large Language Models (LLMs) in preserving nuanced semantic structures and technical terminology in complex academic discourse have been well-documented.
- Robustness Against AI-Generated Content: In the contemporary academic environment, researchers increasingly use AI-mediated translation and paraphrasing tools. Therefore, we intentionally exposed our detection model to these specific "AI artifacts" during the training phase. This ensures that our framework remains robust and effective against modern plagiarism techniques that rely on AI-assisted translations.

By incorporating these translations, we believe our model is better aligned with current real-world challenges in academic integrity.

**7. Data and Code Availability**

**Reviewer Comment: “Dataset and source code are stored in a private GitHub repository... This restricts reproducibility.”**

**Response:**

We fully support the principle of open science. Following the reviewer's and PLOS ONE's requirements, we have made the GitHub repository public. It now includes the full anonymized dataset and the source code required to replicate our findings. [Link: **https://github.com/SaRaABlue/English-Persian-cross-language-plagiarism-detection-multilingual-deep-learning-approach.git**]

**8. Advanced Models and Future Directions**

**Reviewer Comment: “Although the study evaluates multiple machine learning and deep learning models, the focus is primarily on XGBoost and LSTM. Other advanced models, such as transformers or hybrid approaches, could have been explored... The study does not address real-time detection, scalability, or ethics.”**

**Response:**

We sincerely appreciate these forward-looking suggestions. Regarding the exploration of more complex architectures (e.g., further Transformers or hybrid models), we have provided a detailed justification in our Conclusion and Future Work sections.

Our expanded experiments (1,000 samples) revealed a crucial finding: the high-dimensional semantic representations generated by BGE-M3 create a near-linearly separable structure. Consequently, the Logistic Regression (LR) model outperformed more complex architectures like XGBoost and LSTM in terms of stability and generalization (P < 0.05). Since the simpler linear model achieved superior results on the expanded data, we concluded that adding further architectural complexity (like hybrid transformers) would lead to performance saturation without significant gains in accuracy for this specific task.

Regarding the other points mentioned (real-time detection, scalability, and ethics), we have decided to categorize them as Future Research Directions. We believe that addressing these aspects requires a separate, dedicated study for instance, implementing model pruning for real-time deployment or developing Explainable AI (XAI) for ethical justifications. We have expanded our Future Work section to explicitly include these areas (Linguistic Integration, Interpretability & Ethics, Computational Efficiency, and Continuous Learning) to guide future researchers in the field.

**9. Direct Comparison**

**Reviewer Comment: “The study does not provide a direct comparison of its proposed methodology with existing cross-lingual plagiarism detection tools.”**

**Response:**

We acknowledge the reviewer's point regarding the comparison with existing tools. However, most commercial and academic tools for cross-lingual plagiarism detection do not publicly release their underlying models or datasets for the Persian-English pair, making a direct, fair benchmark comparison challenging. To address this, we have prioritized creating a transparent, open-source baseline (now available on GitHub) and have added a discussion in Section 5 regarding the qualitative differences between our approach and existing frameworks.

**10. Applicability to other languages**

**Reviewer Comment: “The study focuses exclusively on Persian-English plagiarism detection, which limits its applicability to other language pairs.”**

**Response:**

We appreciate the reviewer’s comment regarding the focus on the Persian-English pair. Our decision to focus exclusively on this pair was driven by the critical scarcity of high-quality datasets and the limited research available for this specific language combination.

However, we have designed our framework with broader applicability in mind. By selecting the BGE-M3 embedding model, which is specifically engineered for multilingual alignment across a wide range of languages (especially low-resource ones), we have ensured that our methodology is not linguistically "hard-coded." The core pipeline, from embedding generation to classification, is readily adaptable to other language pairs. We have updated our Future Research Directions to explicitly suggest the validation of this framework on additional language pairs as a natural extension of this work.

**Section 5. Conclusion – Page 36– Line 586**

- Cross-Lingual & Disciplinary Expansion: Expanding and validating the framework on a broader range of language pairs (particularly low-resource languages) and diverse scientific disciplines to ensure the global generalizability and robustness of the proposed methodology.

***Response to Reviewer #2***

**Reviewer Comment: “This manuscript addresses a relevant and increasingly important issue... The overall approach is well thought out... The paper is generally well structured, and the experimental workflow is easy to follow.”**

**Response:**

We sincerely thank the reviewer for the positive assessment of our study’s structure and methodology. We are grateful for the recognition of our work’s contribution to the field of academic integrity for low-resource language pairs like Persian-English.

**1. Dataset Realism and Generalizability**

**Reviewer Comment: “The dataset is relatively small and largely based on machine-translated and artificially modified abstracts... Validating the approach on a larger or independent dataset would improve confidence.”**

**Response:**

We appreciate this constructive feedback. We agree that a larger dataset is essential for ensuring the robustness of our results. Following your suggestion, we have expanded our dataset from 501 to 1,000 samples. This expanded set includes a more diverse range of academic abstracts, providing a more reliable basis for our findings. We have re-evaluated all models on this larger dataset, and the updated results (Section 4) demonstrate improved generalizability and performance.

**4.1. Dataset Page 10 – Line 163**

The empirical foundation of this study comprises a curated dataset of 1,000 academic abstracts sourced from the Iranian Research Institute for Information Science and Technology (IranDoc). To facilitate the investigation of cross-lingual plagiarism detection between Persian and English, the original Persian abstracts underwent a systematic translation and modification pipeline. While traditional methodologies have frequently utilized conventional machine translation (MT) engines, this research intentionally employed Microsoft Copilot for the translation phase. This selection was motivated by two primary factors: first, the advanced capability of large language models (LLMs) to preserve nuanced semantic structures in technical discourse [32]; and second, the necessity for the detection model to remain robust against modern, AI-mediated translation artifacts that researchers may encounter in contemporary academic environments.

**2. Robustness Checks and Statistical Significance**

**Reviewer Comment: “The analysis relies solely on descriptive metrics. Including robustness checks such as cross-validation or statistical significance testing would further strengthen the empirical claims.”**

**Response:** We sincerely thank the reviewer for this valuable recommendation. We agree that providing statistical evidence beyond descriptive metrics is crucial for validating the improvements observed in our models.

To strengthen our empirical claims, we performed a McNemar’s Test for statistical significance on the results of the fine-tuned Logistic Regression (LR) model, which emerged as the top performer on the expanded dataset. This test was specifically chosen to determine whether the observed performance gains were a result of genuine model enhancement or merely stochastic variation.

As detailed in the revised Section 4.5 and illustrated in Figure 16, the test yielded an extremely low p-value of 0.00046. This result provides strong empirical evidence that the improvement from the baseline to the fine-tuned LR model is statistically significant and not due to random chance. This rigorous validation reinforces the reliability of our proposed framework and confirms that the optimization process led to a substantial enhancement in detecting cross-lingual plagiarism.

**Section 4.5.2 Fine Tuning for Scaled-Up Classification Page 34 – Line 543**

Statistical Significance Testing

To ensure that the observed performance gains were not a result of stochastic variation or random chance, a McNemar’s Test for statistical significance was performed. This test is specifically suited for comparing the performance of two classifiers on the same dataset. As shown in Fig 16, the test yielded a p-value of 0.00046.

**Fig 16. McNemar’s Test for statistical** **significance**

This extremely low P-value provides strong empirical evidence that the improvement from the baseline to the fine-tuned Logistic Regression model is statistically significant. This result reinforces the reliability of the proposed system, confirming that the optimization process led to a genuine enhancement in the model's ability to detect cross-lingual plagiarism between Persian and English.

**3. Language Polishing and Conciseness**

**Reviewer Comment: “The manuscript would benefit from minor language polishing and some reduction of repetition, particularly in the literature review and results discussion.”**

**Response**:

We appreciate the reviewer's feedback on the manuscript's structure. We have conducted a thorough revision of the text to improve flow and eliminate redundancies, especially in the Literature Review and Discussion sections. We have also addressed minor grammatical issues to ensure the language meets the standard for high-quality scientific publication.

**4. Reproducibility (Data and Code Availability)**

**Reviewer Comment: “The temporary unavailability of the dataset and code also limits reproducibility... making these resources publicly accessible would greatly enhance the value of the work.”**

**Response:** We fully agree that reproducibility is a cornerstone of scientific integrity. In response to your comment, we have made our dataset and complete source code publicly available on GitHub. The link to the repository is now included in the "Data Availability Statement" of the manuscript.

**Data Availability Statement – Page 36 – Line 590**

The dataset and source code underlying the findings of this study have been made publicly available to ensure transparency and reproducibility. All relevant data, including the anonymized academic abstracts and the implementation code for the embedding models and classifiers, can be accessed via the following GitHub repository: [https://github.com/SaRaABlue/English-Persian-cross-language-plagiarism-detection-multilingual-deep-learning-approach.git].

***Response to Reviewer #3***

**Reviewer Comment: “ Overall, this research manuscript offers novel insight... clarifying why XGBoost and LSTM are chosen when the other models seem to perform just as strong if not more so.”**

**Response:**

We thank the reviewer for the positive feedback. Regarding the selection of models, in the initial phase (500 samples), XGBoost and LSTM were prioritized due to their ability to handle non-linear patterns. However, as we expanded the dataset to 1,000 samples and refined the embedding space, our further analysis showed that **Logistic Regression (LR)** actually achieved superior stability and performance. We have updated the manuscript to reflect this shift, justifying why simpler models can be more effective when paired with high-quality embeddings like BGE-M3.

**1. Interpretation of Metrics (Thresholds)**

**Reviewer Comment: “What is the protocol for interpreting recall, accuracy, and F1 scores? In the analysis section, “moderate” is a term to describe SVM’s performance of 80% recall, whereas XGBoost is considered “strong” for 78%...”**

**Response:**

We sincerely thank the reviewer for identifying this inconsistency in our qualitative descriptions. We agree that using terms like "strong" and "moderate" without a predefined threshold can be confusing.

To address this, we have standardized the terminology throughout the revised manuscript based on the following protocol for interpreting performance metrics (Accuracy, Recall, and F1-score):

- **Excellent/Strong:** > 90%
- **Good/High:** 80% - 90%
- **Moderate:** 70% - 80%
- **Weak:** < 70%

Following this framework, we have revised Section 4.4.1 Baseline Classification Using 500 Samples to ensure consistency.

**4.4.1 Baseline Classification Using 500 Samples – Page 18 – Line 302**

In this study, performance metrics are interpreted based on standard NLP benchmarks, where scores above 90% are considered strong, 80-90% as good, and 70-80% as moderate.

The results indicate that most models perform effectively on the Semi-Exact and Different categories, while performance on the Exact class is consistently lower across all methods. In particular, MLP, LSTM, and RNN show similar behavior, achieving good performance in Semi-Exact (up to 80% recall) but lower effectiveness in the Exact category (62% recall, F1 = 0.65). FNN and LR demonstrate comparatively higher discrimination for the Different category, while SVM shows a balanced but overall moderate performance (within the 70-80% range).

**2. Data Accessibility**

**Reviewer Comment: “A possible limitation is that the data is not readily available. Readers must go to a GitHub repository...”**

**Response:**

We understand the concern regarding accessibility. In accordance with PLOS ONE’s open data policy, we have made the GitHub repository Public and ensured it contains the full, anonymized dataset and the complete source code. Furthermore, we have provided a permanent, easy-to-access link in the Data Availability Statement at the end of the manuscript to ensure seamless reproducibility.

**3. Strengthening the Research Gap**

**Reviewer Comment: “Adding specific studies and references to section “2.4 Research gap” will strengthen the section and tie together the thorough background section.”**

**Response:**

We appreciate this constructive suggestion to enhance the academic rigor of our research gap analysis. We have revised Section 2.4 Research gap to more explicitly bridge the findings discussed in the Literature Review with the identified gaps in Persian-English plagiarism detection.

Specifically, we have restructured this section to directly reference the limitations observed in prior studies, such as the reliance on traditional MT engines and the challenges of semantic representation in low-resource settings, which were detailed in our background section. By more tightly integrating these specific references into the Research Gap discussion, we have provided a more cohesive and evidence-based justification for our study's focus on the BGE-M3 model and the necessity of our expanded dataset. This ensures that the identified gaps are clearly supported by the existing body of work discussed earlier in the manuscript.

**2.4.** **Research gap – Page 7 – Line 121**

Table 1 summarizes prior studies. Despite growing advancements in plagiarism detection, research on Persian-English cross-lingual plagiarism remains limited compared to other language pairs. This study addresses several critical gaps identified in the literature:

- Geographic and Linguistic Gap: Most studies focus on European or Arabic-English pairs [5]. While work exists for Persian [31] , there is a lack of high-performance frameworks leveraging recent multilingual deep learning specifically for this pair.
- Semantic Preservation Issues: Existing MT-based methods [6] often fail to capture subtle nuances or struggle with scalability. Even with semantic analysis [5], dependency on traditional translation remains a bottleneck for complex academic texts.
- Overlooking Obfuscation: Much research focuses on verbatim translation, neglecting rewritten or paraphrased plagiarism. Current alignment models often require extensive corpora or complex graphs that are not optimized for low-resource Persian-English data.
- Representation Limitations: Many approaches rely on n-grams [23] or shallow embeddings that ignore word order. This research addresses this by using BGE-M3 encoders to process both languages in a joint semantic space.
- Lack of Statistical Rigor: Studies often report high accuracy but lack rigorous statistical significance testing or large-scale robustness checks. There is a critical need for validated tools that adapt to evolving plagiarism strategies.

**4. Statistical Significance (P-values)**

**Reviewer Comment: “While the fine tuning of the hyperparameters is clearly outlined... it might be helpful to add a more thorough statistical analyses, such as p-values...”**

**Response:**

We are grateful to the reviewer for emphasizing the need for statistical quantification. We agree that P-values provide a more rigorous basis for evaluating the impact of model optimizations. To quantify the benefits of our fine-tuning and optimization process, we have implemented McNemar’s Test to compare the performance of the models before and after optimization. As reported in Section 4.5, the test yielded a p-value of 0.00046, which confirms that the performance improvements are statistically significant (P < 0.05). This addition provides the statistical rigor requested to validate our findings.

**Section 4.5.2 Fine Tuning for Scaled-Up Classification Page 35 – Line 543**

Statistical Significance Testing To ensure that the observed performance gains were not a result of stochastic variation or random chance, a McNemar’s Test for statistical significance was performed. This test is specifically suited for comparing the performance of two classifiers on the same dataset. As shown in Fig 16, the test yielded a p-value of 0.00046.

**Fig 16. McNemar’s Test for statistical** **significance**

This extremely low P-value provides strong empirical evidence that the improvement from the baseline to the fine-tuned Logistic Regression model is statistically significant. This result reinforces the reliability of the proposed system, confirming that the optimization process led to a genuine enhancement in the model's ability to detect cross-lingual plagiarism between Persian and English.

We would like to reiterate our gratitude to the Academic Editor and the three reviewers for their time and effort in evaluating our manuscript. Their constructive criticisms and insightful suggestions have significantly improved the quality, rigor, and clarity of this work.

We believe that the revised manuscript now meets the high standards of PLOS ONE and provides a meaningful contribution to the field of cross-lingual plagiarism detection. We look forward to your positive decision regarding its publication.

Sincerely,

Marzieh Zarinbal, Ph.D.

Assistant Professor

Department of industrial engineering,

Amirkabir University of Technology, Tehran, Iran.

Office: (+9821) 6454

Email: [mzarinbal@aut.ac.ir](mailto:mzarinbal@aut.ac.ir)

Website: <https://scholar.google.com/citations?user=16XztRYAAAAJ&hl=en>
