# Supplementary material for: English-Persian cross-language plagiarism detection: Multilingual deep learning approach
Source: PLoS One. 2026 Sep 30;21(9):e0354459. doi: 10.1371/journal.pone.0354459 (PMC13626316; doi:10.1371/journal.pone.0354459)
Supplement: Supplementary file 3 [file pone.0354459.s004.docx]

**Manuscript Title:** *English-Persian cross-language plagiarism detection: Multilingual deep learning approach*

**Journal:** *PLOS One*
**Manuscript ID:** *PONE-D-25-53532R1*

**Dear Dr. Bolboacă and Reviewers,**

We would like to express our sincere gratitude to the Academic Editor and the Reviewers for their time, constructive feedback, and valuable guidance throughout this review process. We are highly encouraged by the positive remarks from Reviewer 2 and Reviewer 3 regarding the technical soundness, statistical rigor, and open-science transparency of our study.

In accordance with the Academic Editor’s instructions, we have thoroughly updated and reorganized the manuscript to fully align with the scientific writing guidelines and the structural conventions of PLOS ONE. Specifically, we have separated any mixture of methods, results, and discussion into their respective dedicated sections, transformed all bulleted lists into narrative prose, rewritten the introduction, added a comprehensive combined Discussion and Conclusions section, and expanded the methodological details to ensure complete reproducibility.

Below is our point-by-point response to the specific comments raised by the Academic Editor.

**RESPONSE TO ACADEMIC EDITOR**

**ACADEMIC EDITOR Comment 1 *: “Introduction must be start with a critical evaluation of the state of the art not with the gaps.” And “Such paragraphs are not appropriate in scientific writing.”***

**Response:**

We apologize for this structural oversight. In the revised manuscript, the Introduction has been completely rewritten. Specifically, we have implemented the following structural enhancements:

- **Prioritizing the State of the Art:** The opening of the Introduction has been completely rewritten to lead immediately with a critical evaluation of the historical and current state of the art in cross-lingual plagiarism detection. The discussion of existing research gaps has been seamlessly integrated into the later paragraphs to establish a more logical, narrative flow.
- **Removal of the Structural Roadmap:** In accordance with the Editor's precise directive, the inappropriate transitional paragraph outlining the paper's section-by-section organization ("The paper is structured as follows...") has been entirely removed to maintain a concise, formal academic tone.

These extensive improvements have been implemented on Page 3, Line 36.

**Introduction – Page 3, Line 36**

The rapid advancement of machine translation technologies and multilingual large language models (LLMs) has significantly increased the risk of cross-lingual plagiarism, where translated text is presented as original without proper attribution [1]. Compared with monolingual plagiarism detection, cross-lingual detection is more challenging because it requires identifying semantic equivalence across languages with different lexical, syntactic, and morphological structures. As a result, it has become an important area of research for maintaining academic integrity in multilingual environments.

Over the past decade, various approaches have been proposed to address this problem. Early methods relied on machine translation (MT) to convert texts into a single language, followed by traditional monolingual plagiarism detection. More recent studies have focused on cross-lingual alignment techniques, multilingual embeddings, and transformer-based models, which improve semantic matching across languages. However, these methods still depend heavily on high-quality resources and benchmark datasets, and their performance varies across language pairs.

Despite these advances, important challenges remain, especially for low-resource languages such as Persian. The lack of large benchmark datasets and the presence of complex morphological and syntactic structures make reliable detection difficult. In addition, AI-assisted translation tools now generate highly fluent texts that are harder to distinguish from original writing, further increasing the difficulty of detection.

To address these challenges, this study proposes a deep learning framework for Persian–English cross-lingual plagiarism detection. The framework combines multilingual sentence embeddings with supervised classifiers to improve semantic discrimination across plagiarism levels. An AI-assisted benchmark dataset is constructed to reflect modern translation characteristics and varying degrees of similarity. The proposed method is evaluated through comprehensive experiments and statistical analysis.

**ACADEMIC EDITOR Comment 2: *“Write Materials and methods as narrative not as bullet lists.”***

**Response:**

We express our sincere appreciation to the Academic Editor for pointing out this formatting issue. We fully agree that a continuous narrative flow enhances the scholarly quality of the methodology section. In accordance with this guidance, we have meticulously reviewed the entire manuscript, with a particular focus on the "Materials and Methods" section, and completely eliminated all bulleted, formatted, or numbered lists, converting them into cohesive, academic prose throughout. To demonstrate these structural improvements, we have provided a few representative examples of the revisions below.

**Research gap – Page 7, Line 127**

Table 1 summarizes prior studies. Regarding the Geographic and Linguistic Gap, most studies focus on European or Arabic-English pairs [6]. While work exists for Persian [16], there is a lack of high-performance frameworks leveraging recent multilingual deep learning specifically for this pair. In terms of Semantic Preservation Issues, existing MT-based methods [7] often fail to capture subtle nuances or struggle with scalability. Even with semantic analysis [5], dependency on traditional translation remains a bottleneck for complex academic texts.

Furthermore, addressing the problem of Overlooking Obfuscation, much research focuses on verbatim translation, neglecting rewritten or paraphrased plagiarism. Current alignment models often require extensive corpora or complex graphs that are not optimized for low-resource Persian-English data. Concerning Representation Limitations, many approaches rely on n-grams [28] or shallow embeddings that ignore word order. This research addresses this by using BGE-M3 encoders to process both languages in a joint semantic space. Finally, regarding the Lack of Statistical Rigor, studies often report high accuracy but lack rigorous statistical significance testing or large-scale robustness checks. There is a critical need for validated tools that adapt to evolving plagiarism strategies.

**Materials & methods – Page 9, Line 144**

This study follows a methodological framework that outlines the key steps undertaken in the research process. The dataset consists of the abstracts of Persian scientific documents along with their English translations, and to enhance the diversity of the dataset, two versions, semi‑exact and different, were generated by applying semantic and structural transformations to the original abstracts.

For text representation and similarity measurement, the BAAI/BGE‑M3 model is utilized to convert abstracts into vector representations, after which the cosine similarity between Persian and English abstracts is calculated to assess their resemblance. Based on the obtained similarity scores, the abstracts are labeled into predefined categories, and the dataset is subsequently divided into training and testing subsets.

In the classification phase, various classification algorithms are applied and evaluated using accuracy, recall, and F1‑score to determine their effectiveness. Finally, the best‑performing models undergo fine‑tuning and further evaluation to enhance their performance. By following this methodology, we ensure a robust approach to detecting cross‑lingual plagiarism between Persian and English texts. Fig 1 visually represents the framework, from data preparation to model evaluation.

**Page 11, Line 178**

To systematically augment the text, two primary paraphrasing techniques were employed. First, synonym substitution was conducted by replacing specific words with their appropriate synonyms utilizing the NLTK library and the WordNet database [30]. Second, sentence reordering was applied to alter sentence structures while carefully preserving the overall contextual meaning. Since structural modifications could potentially impact semantic coherence, manual supervision was subsequently performed to guarantee the logical consistency of the generated text [31].

**Page 16 – Line 276**

To optimize detection across varying degrees of similarity, the framework establishes distinct coverage boundaries using specialized classifiers. For lower-bound coverage targeting exact categories, XGBoost was selected due to its superior ability to capture direct translations, achieving the highest recall of 0.84 and ensuring that verbatim theft is identified with high sensitivity. Conversely, for upper-bound coverage across different categories, Logistic Regression was utilized because of its peak performance in the most complex classes, yielding a maximum recall of 0.91 and providing a robust defense against sophisticated, multi-source plagiarism.

**Page 23 - Line 388**

The expansion of the dataset to 1,000 samples has significantly stabilized the models, allowing for a clearer differentiation of the discriminative power of each algorithm. Regarding recurrent and sequence models such as LSTM and RNN, these architectures exhibited high stability, particularly in the Semi-Exact category, where LSTM maintained a balanced F1-score of 0.92, thereby confirming the efficacy of gated architectures in capturing semantic dependencies. However, their sensitivity in the Exact class was slightly lower than that of ensemble methods. In comparison, Support Vector Machines (SVM) showed the highest Recall in the Semi-Exact category (0.94), but its performance in the Exact category was the second-lowest (0.68), suggesting a potential for over-generalization in detecting direct translations. Meanwhile, Logistic Regression (LR) demonstrated exceptional robustness in identifying complex plagiarism, notably achieving the highest Recall in the Different category (0.91) and maintaining top-tier Precision (0.90) in the Exact class. Additionally, the XGBoost Classifier proved to be the most effective for detecting near-verbatim plagiarism, achieving the peak Recall of 0.84 in the Exact category, which represents a significant improvement over the previous iteration. Based on the multi-dimensional assessment of these metrics, XGBoost and Logistic Regression were strategically selected for subsequent hyperparameter fine-tuning and optimization.

**Academic Editor Comment 3: *“4. Results and Evaluation is a mixture of methods, results and discussion. Move text to appropriate sections. The Methods should be presented in sufficient details to allow reproduction/replication.”***

**Response:**

We sincerely thank the Academic Editor for this highly constructive recommendation. We completely agree that a clear separation between experimental procedures, data reporting, and analysis is essential for academic clarity and scientific replication. In accordance with this valuable guidance, we have carefully reorganized the manuscript to ensure each component resides in its proper section:

- Methodological Depth (Section 3 - Materials and Methods): We have relocated all procedural details, hyperparameter configurations, and the statistical framework into Section 3. We hope this comprehensive description now provides all the necessary detail to facilitate straightforward reproduction of our work. ***(Start from Page 10, Line 161- Dataset)***
- Pure Data Reporting (Section 4 - Results): This section has been streamlined to focus exclusively on presenting our empirical outcomes, performance metrics, and tables, completely free of any methodological or interpretive text.***(Start from Page 18, Line 312 – Results)***

Additionally, to further support transparency and replication as kindly noted and appreciated by ***Reviewer #2***, the complete source code and anonymized dataset remain fully accessible through our public repository.

All of these structural improvements and text relocations have been clearly highlighted in the revised manuscript to make your review as convenient as possible.

***Academic Editor Comment 3: “Discussion section is missing.”***

**Response:**

We are very grateful to the Academic Editor for this valuable and constructive suggestion. We appreciate the opportunity to further elaborate on our findings, and we agree that incorporating a dedicated section significantly enhances the depth, contextual interpretation, and overall clarity of our manuscript.

To address this and satisfy the structural criteria of PLOS ONE, we have extensively revised the manuscript by introducing a standalone section titled "Discussion". This new section offers a rigorous, multi-faceted interpretation of our empirical results. Specifically, it provides an in-depth algorithmic explanation of why the linear model performed robustly compared to more complex networks, analyzed through both high-dimensional vector geometry (the 1024-dimensional semantic space) and cross-lingual linguistic structures, such as the syntactic alignment between Persian (SOV) and English (SVO).

Concurrently, we have refined the subsequent "Conclusions" section to ensure clarity and eliminate any thematic overlap with the discussion. In strict adherence to the journal's submission guidelines, we have carefully polished this section to summarize our core findings without overstating their impact. Furthermore, the future research directions, which were previously formatted as bullet points, have been completely converted into continuous, narrative prose. We believe these structural improvements significantly enhance the academic rigor and readability of our work.

**Discussion – Page 33, Line 560**

From an algorithmic perspective, the superior performance of Logistic Regression (LR) over non-linear models like XGBoost and LSTM suggests that the semantic embedding space generated by BGE-M3 may exhibit a relatively high degree of linear separability for this task. The BGE-M3 model projects text into a dense, 1024-dimensional semantic space, where semantically similar documents are positioned closer together. Within this embedding space, LR appears to construct an effective decision boundary that generalizes well while maintaining low model complexity. In contrast, XGBoost may derive less benefit from recursive feature partitioning in dense continuous embedding spaces, leading to limited performance gains over simpler linear classifiers. Similarly, because BGE-M3 compresses contextual and sequential information into a single fixed-length semantic representation, the sequential modeling capability of LSTM may provide limited additional discriminative information. These findings suggest that, for low-resource cross-lingual plagiarism detection, the quality of semantic representations may play a more important role than increasing downstream classifier complexity.

Linguistically, these findings suggest that the embedding model effectively mitigates syntactic disparities between Persian (Subject–Object–Verb) and English (Subject–Verb–Object). While conventional classifiers often struggle to bridge these cross-lingual differences with limited training data, BGE-M3 appears to map semantically equivalent concepts into a shared representation space during pre-training, regardless of surface-level linguistic structure. As a result, downstream classification becomes largely dependent on semantic proximity within the embedding space, enabling LR to effectively discriminate between plagiarism categories without relying on complex non-linear feature interactions.

Furthermore, expanding the dataset from 500 to 1,000 samples improved model stability and reduced ambiguity between the Exact and Semi-Exact plagiarism classes. However, the persistent confusion between these categories underscores the ongoing technical challenge of distinguishing literal translations from highly faithful paraphrases. These findings are consistent with previous studies highlighting the advantages of semantic embedding models over conventional translation-then-monolingual pipelines. Moreover, the results suggest that carefully optimized conventional machine learning classifiers can achieve performance comparable to more complex deep learning architectures when supported by high-quality multilingual semantic representations, while offering lower computational complexity and greater interpretability.

**Conclusions– Page 36 , Line 591**

This study investigated cross-lingual plagiarism detection in Persian–English academic abstracts, specifically addressing the challenges posed by machine translation and complex paraphrasing. By leveraging the BGE-M3 embedding model and expanding the dataset to 1,000 samples, the empirical results demonstrated that downstream classifier selection becomes increasingly important as dataset size increases. A key finding of this research is the superior generalization performance of the optimized Logistic Regression (LR) model on the expanded dataset. While XGBoost and LSTM showed strong performance on the initial 500-sample dataset, the optimized LR model achieved the best overall performance on the larger dataset. Ultimately, the proposed framework provides a computationally efficient and scalable solution for cross-lingual plagiarism detection in academic publishing systems.

However, the consistent difficulty in identifying the Exact plagiarism category across all models, attributed to the extreme semantic proximity between verbatim and near-verbatim translations, remains a key technical challenge that warrants further methodological investigation.

To further enhance the effectiveness and reliability of cross-lingual plagiarism detection, future research should explore the integration of syntactic information, Named Entity Recognition (NER), and authorship-related metadata to improve plagiarism detection performance. Developing Explainable AI (XAI) techniques could provide transparent justifications for plagiarism decisions, increase user trust, and reduce the impact of false-positive detections. From a practical perspective, applying model pruning and quantization may improve computational efficiency and facilitate real-time, large-scale deployment in academic publishing workflows. In addition, incorporating continual learning mechanisms would enable plagiarism detection systems to adapt to evolving language patterns and emerging AI-assisted plagiarism strategies

Finally, future studies should investigate class imbalance mitigation techniques, including oversampling and class-weighted loss functions, to improve model reliability in real-world environments where genuine documents substantially outnumber plagiarized ones. Expanding the proposed framework to larger and more diverse datasets spanning multiple scientific disciplines and additional cross-lingual settings, particularly low-resource language pairs, would further improve the generalizability, robustness, and practical applicability of the proposed methodology.

We would like to conclude by reiterating our sincere gratitude to the Academic Editor, Dr. Bolboacă, for providing such precise structural guidance, as well as to Reviewer #2 and Reviewer #3 for their valuable time, thorough evaluations, and encouraging support of our study. The manuscript has been deeply refined, and we firmly believe that these collaborative efforts have significantly improved its clarity, academic organization, and adherence to the high standards of PLOS ONE.

Sincerely,

Marzieh Zarinbal, Ph.D.

Assistant Professor

Department of industrial engineering,

Amirkabir University of Technology, Tehran, Iran.

Office: (+9821) 6454

Email: [mzarinbal@aut.ac.ir](mailto:mzarinbal@aut.ac.ir)

Website: <https://scholar.google.com/citations?user=16XztRYAAAAJ&hl=en>
