# Supplementary material for: English-Persian cross-language plagiarism detection: Multilingual deep learning approach
Source: PLoS One. 2026 Sep 30;21(9):e0354459. doi: 10.1371/journal.pone.0354459 (PMC13626316; doi:10.1371/journal.pone.0354459)
Supplement: Peer review history

# Peer review: Decision Letter 0

**DOI:** 10.1371/journal.pone.0354459.r001

**Authors:** Sorana Bolboacă

23 Feb 2026

Dear Dr. Zarinbal,

Thank you for submitting your manuscript to PLOS ONE. After careful consideration, we feel that it has merit but does not fully meet PLOS ONE’s publication criteria as it currently stands. Therefore, we invite you to submit a revised version of the manuscript that addresses the points raised during the review process.

==============================

**ACADEMIC EDITOR:**

- Please carefully address all reviewer's comments and suggestions.

Please submit your revised manuscript by Apr 09 2026 11:59PM. If you will need more time than this to complete your revisions, please reply to this message or contact the journal office at plosone@plos.org. When you're ready to submit your revision, log on to https://www.editorialmanager.com/pone/ and select the 'Submissions Needing Revision' folder to locate your manuscript file.

- A letter that responds to each point raised by the academic editor and reviewer(s). You should upload this letter as a separate file labeled 'Response to Reviewers'.
- A marked-up copy of your manuscript that highlights changes made to the original version. You should upload this as a separate file labeled 'Revised Manuscript with Track Changes'.
- An unmarked version of your revised paper without tracked changes. You should upload this as a separate file labeled 'Manuscript'.

If applicable, we recommend that you deposit your laboratory protocols in protocols.io to enhance the reproducibility of your results. Protocols.io assigns your protocol its own identifier (DOI) so that it can be cited independently in the future. For instructions see: https://journals.plos.org/plosone/s/submission-guidelines#loc-laboratory-protocols. Additionally, PLOS ONE offers an option for publishing peer-reviewed Lab Protocol articles, which describe protocols hosted on protocols.io. Read more information on sharing protocols at https://plos.org/protocols?utm_medium=editorial-email&utm_source=authorletters&utm_campaign=protocols.

We look forward to receiving your revised manuscript.

Kind regards,

Sorana D. Bolboacă, Ph.D., M.Sc., M.D.

Academic Editor

PLOS One

Journal Requirements:

When submitting your revision, we need you to address these additional requirements.

1.Please ensure that your manuscript meets PLOS ONE's style requirements, including those for file naming. The PLOS ONE style templates can be found at

https://journals.plos.org/plosone/s/file?id=wjVg/PLOSOne_formatting_sample_main_body.pdf and

https://journals.plos.org/plosone/s/file?id=ba62/PLOSOne_formatting_sample_title_authors_affiliations.pdf

2. Please note that PLOS One has specific guidelines on code sharing for submissions in which author-generated code underpins the findings in the manuscript. In these cases, we expect all author-generated code to be made available without restrictions upon publication of the work. Please review our guidelines at https://journals.plos.org/plosone/s/materials-and-software-sharing#loc-sharing-code and ensure that your code is shared in a way that follows best practice and facilitates reproducibility and reuse.

3. We note that you have indicated that there are restrictions to data sharing for this study. PLOS only allows data to be available upon request if there are legal or ethical restrictions on sharing data publicly. For more information on unacceptable data access restrictions, please see http://journals.plos.org/plosone/s/data-availability#loc-unacceptable-data-access-restrictions.

Before we proceed with your manuscript, please address the following prompts:

a) If there are ethical or legal restrictions on sharing a de-identified data set, please explain them in detail (e.g., data contain potentially identifying or sensitive patient information, data are owned by a third-party organization, etc.) and who has imposed them (e.g., a Research Ethics Committee or Institutional Review Board, etc.). Please also provide contact information for a data access committee, ethics committee, or other institutional body to which data requests may be sent.

b) If there are no restrictions, please upload the minimal anonymized data set necessary to replicate your study findings to a stable, public repository and provide us with the relevant URLs, DOIs, or accession numbers. For a list of recommended repositories, please see

https://journals.plos.org/plosone/s/recommended-repositories. You also have the option of uploading the data as Supporting Information files, but we would recommend depositing data directly to a data repository if possible.

We will update your Data Availability statement on your behalf to reflect the information you provide.

4. When completing the data availability statement of the submission form, you indicated that you will make your data available on acceptance. We strongly recommend all authors decide on a data sharing plan before acceptance, as the process can be lengthy and hold up publication timelines. Please note that, though access restrictions are acceptable now, your entire data will need to be made freely accessible if your manuscript is accepted for publication. This policy applies to all data except where public deposition would breach compliance with the protocol approved by your research ethics board. If you are unable to adhere to our open data policy, please kindly revise your statement to explain your reasoning and we will seek the editor's input on an exemption. Please be assured that, once you have provided your new statement, the assessment of your exemption will not hold up the peer review process.

5. If the reviewer comments include a recommendation to cite specific previously published works, please review and evaluate these publications to determine whether they are relevant and should be cited. There is no requirement to cite these works unless the editor has indicated otherwise.

6. Please review your reference list to ensure that it is complete and correct. If you have cited papers that have been retracted, please include the rationale for doing so in the manuscript text, or remove these references and replace them with relevant current references. Any changes to the reference list should be mentioned in the rebuttal letter that accompanies your revised manuscript. If you need to cite a retracted article, indicate the article’s retracted status in the References list and also include a citation and full reference for the retraction notice.

[Note: HTML markup is below. Please do not edit.]

Reviewer's Responses to Questions

**Comments to the Author**

1. Is the manuscript technically sound, and do the data support the conclusions?

Reviewer #1: Yes

Reviewer #2: Partly

Reviewer #3: Yes

**********

2. Has the statistical analysis been performed appropriately and rigorously?

Reviewer #1: Yes

Reviewer #2: Yes

Reviewer #3: No

**********

3. Have the authors made all data underlying the findings in their manuscript fully available?

The PLOS Data policy

Reviewer #1: Yes

Reviewer #2: No

Reviewer #3: No

**********

4. Is the manuscript presented in an intelligible fashion and written in standard English?

Reviewer #1: Yes

Reviewer #2: Yes

Reviewer #3: Yes

**********

Reviewer #1: The theme of the article—English-Persian cross-language plagiarism detection using a multilingual deep learning approach—sound and is relevant and timely. The article presents a novel approach to English-Persian cross-language plagiarism detection using advanced machine learning and deep learning techniques. With the increasing prevalence of academic misconduct and the growing use of translation tools, addressing cross-lingual plagiarism is a critical issue. The focus on Persian-English plagiarism detection fills a notable research gap, as most existing studies have concentrated on other language pairs. The theme is well-justified and aligns with the broader goals of promoting academic integrity and research ethics.

The scientific language used in the article is appropriate for a research paper. The authors employ technical terminology relevant to the fields of natural language processing (NLP), machine learning, and plagiarism detection, such as "cosine similarity," "embedding models," "classification algorithms," and "fine-tuning." The explanations of methods, such as BERT and BGE-M3 embeddings, are detailed and clear, making the paper accessible to readers with a background in machine learning or computational linguistics.

However, there are instances where the language could be more concise or structured for clarity. For example, the introduction and conclusion sections seem very detailed, which may dilute the impact of the key findings.Some technical terms, such as "contrastive distance," are introduced without sufficient explanation for readers unfamiliar with the concept.

There are no explicit data errors in the article, as the authors provide detailed descriptions of their dataset, methods, and evaluation metrics. However, there are some concerns:

The dataset consists of only 501 abstracts, which is relatively small for training and testing machine learning models. This could lead to biased or overfitted results, especially for complex tasks like cross-lingual plagiarism detection.

The dataset is evenly distributed across three categories (Exact, Semi-Exact, Different), which may not reflect real-world scenarios where plagiarism cases are often imbalanced. This could affect the model's performance in practical applications.

The correlations and conclusions drawn in the study appear to be logical and supported by the data.

The authors use appropriate evaluation metrics (accuracy, precision, recall, and F1-score) to assess the performance of their models. However, there are some areas that could be improved: The authors conclude that BGE-M3 embeddings outperform BERT for Persian-English text pairs. While this conclusion is supported by the data, the study does not explore why BERT underperformed or whether additional fine-tuning could improve its results. Need more specification the correlation Between Embedding Models and Performance

The study highlights that XGBoost and LSTM performed better than other models, but the Exact category consistently showed lower recall and F1 scores. The authors attribute this to inter-class similarities but do not explore alternative explanations or potential improvements for this issue.

While the study demonstrates promising results, however some fragility can be identified:

The dataset used in the study was limited due consists of only 501 abstracts, which may not be sufficient to ensure the generalizability and robustness of the model. A larger and more diverse dataset, including texts from various disciplines, would improve the reliability of the results.

The study focuses exclusively on Persian-English plagiarism detection, which limits its applicability to other language pairs. Expanding the approach to additional languages could enhance its utility and impact.

Both XGBoost and LSTM models struggled with the Exact category, showing lower recall and F1 scores compared to the Semi-Exact and Different categories. This indicates that the models may not be fully optimized for detecting direct translations or detecting exact plagiarism

The study relies heavily on Microsoft Copilot for translation, which may introduce biases or errors inherent in the tool. The accuracy of the plagiarism detection system is contingent on the quality of the translations, which could vary depending on the text complexity.

Although the authors mention that the data is available on demand, there is an indication that dataset and source code are stored in a private GitHub repository and are not publicly available. This restricts reproducibility and transparency, which are critical for validating the study's findings and enabling further research.

While the study proposes a robust framework, it does not address the development of real-time plagiarism detection systems. Real-time tools are essential for practical applications in academic and publishing environments.

Although the study evaluates multiple machine learning and deep learning models, the focus is primarily on XGBoost and LSTM. Other advanced models, such as transformers or hybrid approaches, could have been explored for potentially better results.

The study mentions efforts to mitigate overfitting during fine-tuning, but the relatively small dataset size and the complexity of the models used may still pose risks of overfitting, which could affect the model's performance on unseen data.

The study does not provide a direct comparison of its proposed methodology with existing cross-lingual plagiarism detection tools. This makes it difficult to assess the relative performance and advantages of the proposed approach.

While the study discusses the importance of academic integrity, it does not address potential ethical concerns or limitations of using AI-powered tools for plagiarism detection, such as false positives or the implications of automated decision-making. Addressing these problems in future research could enhance the effectiveness, applicability, and reliability of the proposed methodology.

The study focuses on embeddings and classification models but does not explore other features that could improve detection accuracy as contextual features like named entity recognition, syntactic structures, and semantic roles to enhance detection. Other example that could be taken is the metadata analysis (e.g., publication dates, authorship patterns) to identify potential plagiarism cases.

The dataset used in the study is evenly distributed across three categories (Exact, Semi-Exact, Different), which may not reflect real-world scenarios.

Use of techniques to imbalanced dataset handling like oversampling, undersampling, or class-weighted loss functions to address class imbalance in real-world applications.

The study does not discuss also:

- The scalability and Computational Efficiency of the proposed system for large-scale applications. Explore optimization for large datasets methods to reduce computational overhead, such as model pruning or quantization;

- Investigate the feasibility of deploying the system on cloud platforms integration for real-time detection.

The study does not address how the system can adapt to evolving language patterns and new plagiarism techniques. In case of continuous learning: implement mechanisms for the model to learn from new data and improve over time. Must refer the importance of regularly update the system to account, monitoring trends for changes in language use and emerging plagiarism strategies.

Reviewer #2: This manuscript addresses a relevant and increasingly important issue in academic integrity, particularly for low-resource language pairs such as Persian–English. The overall approach is well thought out, and the combination of multilingual sentence embeddings with machine learning and deep learning classifiers is appropriate for the problem being studied. The paper is generally well structured, and the experimental workflow—from dataset construction to model evaluation and fine-tuning—is easy to follow.

The results are clearly presented and show that the proposed method, especially when using XGBoost and LSTM with fine-tuning, can improve the detection of cross-lingual plagiarism. The use of multiple evaluation metrics and confusion matrices helps the reader understand the strengths and limitations of the models across different plagiarism categories.

There are, however, some areas where the study could be strengthened. The dataset is relatively small and largely based on machine-translated and artificially modified abstracts, which may not fully capture the complexity of real-world plagiarism cases. Validating the approach on a larger or independent dataset would improve confidence in the generalizability of the findings. In addition, while performance improvements are reported clearly, the analysis relies solely on descriptive metrics. Including robustness checks such as cross-validation or statistical significance testing would further strengthen the empirical claims.

Finally, the manuscript would benefit from minor language polishing and some reduction of repetition, particularly in the literature review and results discussion. The temporary unavailability of the dataset and code also limits reproducibility, and making these resources publicly accessible upon publication would greatly enhance the value of the work.

The study is solid and promising study, and with these refinements, it has the potential to make a meaningful contribution to research on cross-lingual plagiarism detection.

Reviewer #3: Overall, this research manuscript offers novel insight as to how machine learning can address a gap in cross-cultural communication and plagiarism detection. While the background information section is detailed and helps contextualize the research, perhaps clarifying why XGBoost and LSTM are chosen when the other models seem to perform just as strong if not more so.

Major Comments:

What is the protocol for interpreting recall, accuracy, and F1 scores? What threshold is considered a strong or weak score? In the analysis section, “moderate” is a term to describe SVM’s performance of 80% recall, whereas XGBoost is considered “strong” for 78% in the next paragraph. Clarifying how these metrics are intended to be interpreted might be helpful for the reader in understanding the results.

A possible limitation is that the data is not readily available. Readers must go to a GitHub repository to access the data for this research. Ensuring that this data is more easily accessible could help with reproducibility.

Adding specific studies and references to section “2.4 Research gap” will strengthen the section and tie together the thorough background section.

While the fine tuning of the hyperparameters is clearly outlined in the methods section, it might be helpful to add a more thorough statistical analyses, such as p-values, to help the reader quantify the benefit of these adjustments.

**********

what does this mean?). If published, this will include your full peer review and any attached files.

If you choose “no”, your identity will remain anonymous but your review may still be made public.

**Do you want your identity to be public for this peer review?**  For information about this choice, including consent withdrawal, please see our Privacy Policy

Reviewer #1: No

Reviewer #2: No

Reviewer #3: **Yes:** Paula Beatty

**********

[NOTE: If reviewer comments were submitted as an attachment file, they will be attached to this email and accessible via the submission site. Please log into your account, locate the manuscript record, and check for the action link "View Attachments". If this link does not appear, there are no attachment files.]

To ensure your figures meet our technical requirements, please review our figure guidelines: https://journals.plos.org/plosone/s/figures

You may also use PLOS’s free figure tool, NAAS, to help you prepare publication quality figures: https://journals.plos.org/plosone/s/figures#loc-tools-for-figure-preparation.

NAAS will assess whether your figures meet our technical requirements by comparing each figure against our figure specifications.

Attachment Submitted filename: Beatty Review for PLOS PONE-D-25-53532.pdf

# Author comment: Author response to Decision Letter 1

**DOI:** 10.1371/journal.pone.0354459.r002

16 May 2026

Thank you for your email and for the opportunity to submit a revised version of our manuscript. We appreciate the constructive feedback provided by you and the reviewers, which has helped us significantly improve the quality and robustness of our study.

We have addressed all the comments raised by the reviewers and the editorial requirements. Most notably, we have:

• Expanded our dataset from 501 to 1,000 samples to ensure better generalizability.

• Included statistical significance testing (McNemar’s test) to validate the model comparisons.

• Made our dataset and source code publicly available on GitHub to comply with PLOS ONE’s data availability policy.

• Refined the manuscript's structure for better conciseness, particularly in the Introduction, Classification and Conclusion sections.

Attachment Submitted filename: Response Letter.docx

# Peer review: Decision Letter 1

**DOI:** 10.1371/journal.pone.0354459.r003

**Authors:** Sorana Bolboacă

26 Jun 2026

Dear Dr. Zarinbal,

Thank you for submitting your manuscript to PLOS ONE. After careful consideration, we feel that it has merit but does not fully meet PLOS ONE’s publication criteria as it currently stands. Therefore, we invite you to submit a revised version of the manuscript that addresses the points raised during the review process.

**ACADEMIC EDITOR:**

- Introduction must be start with a critical evaluation of the state of the art not with the gaps.
- "The paper is structured as follows: Section 2 critically reviews existing literature and identifies the research gap; Section 3 details the proposed methodology and architectural design; Section 4
  presents the experimental evaluation and statistical validation; and Section 5 concludes the study
  with a discussion on broader implications and future research directions." Such paragraphs are not appropriate in scientific writing.
- Present the contributions to Discussion section.
- Write Materials and methods as narrative not as bullet lists.
- "4. Results and Evaluation" is a mixture of methods, results and discussion. Move text to appropriate sections.
- The Methods should be presented in sufficient details to allow reproduction/replication.
- Discussion section is missing.
- Provide specific feedback from your evaluation of the manuscript

Please submit your revised manuscript by Aug 10 2026 11:59PM. If you will need more time than this to complete your revisions, please reply to this message or contact the journal office at plosone@plos.org. When you're ready to submit your revision, log on to https://www.editorialmanager.com/pone/ and select the 'Submissions Needing Revision' folder to locate your manuscript file.

- A letter that responds to each point raised by the academic editor and reviewer(s). You should upload this letter as a separate file labeled 'Response to Reviewers'.
- A marked-up copy of your manuscript that highlights changes made to the original version. You should upload this as a separate file labeled 'Revised Manuscript with Track Changes'.
- An unmarked version of your revised paper without tracked changes. You should upload this as a separate file labeled 'Manuscript'.

If you would like to make changes to your financial disclosure, please include your updated statement in your cover letter. Guidelines for resubmitting your figure files are available below the reviewer comments at the end of this letter.

If applicable, we recommend that you deposit your laboratory protocols in protocols.io to enhance the reproducibility of your results. Protocols.io assigns your protocol its own identifier (DOI) so that it can be cited independently in the future. For instructions see: https://journals.plos.org/plosone/s/submission-guidelines#loc-laboratory-protocols. Additionally, PLOS ONE offers an option for publishing peer-reviewed Lab Protocol articles, which describe protocols hosted on protocols.io. Read more information on sharing protocols at https://plos.org/protocols?utm_medium=editorial-email&utm_source=authorletters&utm_campaign=protocols.

As the corresponding author, your ORCID iD is verified in the submission system and will appear in the published article. PLOS supports the use of ORCID, and we encourage all coauthors to register for an ORCID iD and use it as well. Please encourage your coauthors to verify their ORCID iD within the submission system before final acceptance, as unverified ORCID iDs will not appear in the published article. *Only*  the individual author can complete the verification step; PLOS staff *cannot*  verify ORCID iDs on behalf of authors.

We look forward to receiving your revised manuscript.

Kind regards,

Sorana D. Bolboacă, Ph.D., M.Sc., M.D.

Academic Editor

PLOS One

**Journal Requirements:**

If the reviewer comments include a recommendation to cite specific previously published works, please review and evaluate these publications to determine whether they are relevant and should be cited. There is no requirement to cite these works unless the editor has indicated otherwise.

[Note: HTML markup is below. Please do not edit.]

Reviewers' comments:

Reviewer's Responses to Questions

**Comments to the Author**

Reviewer #2: All comments have been addressed

Reviewer #3: All comments have been addressed

**********

2. Is the manuscript technically sound, and do the data support the conclusions?

Reviewer #2: Yes

Reviewer #3: Yes

**********

3. Has the statistical analysis been performed appropriately and rigorously?

Reviewer #2: Yes

Reviewer #3: Yes

**********

4. Have the authors made all data underlying the findings in their manuscript fully available?

The PLOS Data policy

Reviewer #2: Yes

Reviewer #3: Yes

**********

5. Is the manuscript presented in an intelligible fashion and written in standard English?

Reviewer #2: Yes

Reviewer #3: Yes

**********

Reviewer #2: I appreciated the responses maid by the authors to my previous comments. The revisions made have significantly strengthened the manuscript, addressing the core concerns regarding dataset realism, generalizability, and statistical validation.

In particular, I appreciate the following improvements:

Dataset Expansion: Expanding the dataset from 501 to 1,000 samples substantially enhances the reliability and generalizability of the empirical findings. Using an LLM (Microsoft Copilot) to simulate modern, AI-mediated translation artifacts is a well-justified approach that reflects contemporary challenges in academic integrity.

Statistical Rigor: The inclusion of McNemar's test provides the necessary statistical validation to confirm that the performance leap of the fine-tuned Logistic Regression model (96% accuracy, p < 0.00046) is genuine and not a result of stochastic variation.

Transparency and Open Science:Making the author-generated code and the anonymized dataset publicly available on GitHub aligns perfectly with the journal's data availability policies and ensures reproducibility.

The manuscript is now well-structured, clear, and makes a valuable contribution to cross-lingual plagiarism detection for low-resource language pairs. I am satisfied with the revisions and recommend the paper for publication.

Reviewer #3: (No Response)

**********

what does this mean?). If published, this will include your full peer review and any attached files.

If you choose “no”, your identity will remain anonymous but your review may still be made public.

**Do you want your identity to be public for this peer review?**  For information about this choice, including consent withdrawal, please see our Privacy Policy

Reviewer #2: No

Reviewer #3: No

**********

[NOTE: If reviewer comments were submitted as an attachment file, they will be attached to this email and accessible via the submission site. Please log into your account, locate the manuscript record, and check for the action link "View Attachments". If this link does not appear, there are no attachment files.]

To ensure your figures meet our technical requirements, please review our figure guidelines: https://journals.plos.org/plosone/s/figures

You may also use PLOS’s free figure tool, NAAS, to help you prepare publication quality figures: https://journals.plos.org/plosone/s/figures#loc-tools-for-figure-preparation.

NAAS will assess whether your figures meet our technical requirements by comparing each figure against our figure specifications.

# Author comment: Author response to Decision Letter 2

**DOI:** 10.1371/journal.pone.0354459.r004

2 Jul 2026

Dear Dr. Bolboacă and Reviewers,

We would like to express our sincere gratitude to the Academic Editor and the Reviewers for their time, constructive feedback, and valuable guidance throughout this review process. We are highly encouraged by the positive remarks from Reviewer 2 and Reviewer 3 regarding the technical soundness, statistical rigor, and open-science transparency of our study.

In accordance with the Academic Editor’s instructions, we have thoroughly updated and reorganized the manuscript to fully align with the scientific writing guidelines and the structural conventions of PLOS ONE. Specifically, we have separated any mixture of methods, results, and discussion into their respective dedicated sections, transformed all bulleted lists into narrative prose, rewritten the introduction, added a comprehensive combined Discussion and Conclusions section, and expanded the methodological details to ensure complete reproducibility.

The point-by-point response to the specific comments raised by the Academic Editor was answered in Response Letter.

Sincerely,

Marzieh Zarinbal, Ph.D.

Assistant Professor

Department of industrial engineering,

Amirkabir University of Technology, Tehran, Iran.

Office: (+9821) 6454

Email: mzarinbal@aut.ac.ir

Website: https://scholar.google.com/citations?user=16XztRYAAAAJ&hl=en

Attachment Submitted filename: English-Persian plagiarism-Rev. V02-Response Letter.docx

# Editor report: Decision Letter 2

**DOI:** 10.1371/journal.pone.0354459.r005

**Authors:** Sorana Bolboacă

9 Jul 2026

English-Persian cross-language plagiarism detection: multilingual deep learning approach

PONE-D-25-53532R2

Dear Dr. Zarinbal,

We’re pleased to inform you that your manuscript has been judged scientifically suitable for publication and will be formally accepted for publication once it meets all outstanding technical requirements.

Within one week, you’ll receive an e-mail detailing the required amendments. When these have been addressed, you’ll receive a formal acceptance letter and your manuscript will be scheduled for publication.

An invoice will be generated when your article is formally accepted. Please note, if your institution has a publishing partnership with PLOS and your article meets the relevant criteria, all or part of your publication costs will be covered. Please make sure your user information is up-to-date by logging into Editorial Manager at Editorial Manager® and clicking the ‘Update My Information' link at the top of the page. For questions related to billing, please contact billing support.

If your institution or institutions have a press office, please notify them about your upcoming paper to help maximize its impact. If they’ll be preparing press materials, please inform our press team as soon as possible -- no later than 48 hours after receiving the formal acceptance. Your manuscript will remain under strict press embargo until 2 pm Eastern Time on the date of publication. For more information, please contact onepress@plos.org.

Kind regards,

Sorana D. Bolboacă, Ph.D., M.Sc., M.D.

Academic Editor

PLOS One

# Editor report: Acceptance letter

**DOI:** 10.1371/journal.pone.0354459.r006

**Authors:** Sorana Bolboacă

PONE-D-25-53532R2

PLOS One

Dear Dr. Zarinbal,

I'm pleased to inform you that your manuscript has been deemed suitable for publication in PLOS One. Congratulations! Your manuscript is now being handed over to our production team.

At this stage, our production department will prepare your paper for publication. This includes ensuring the following:

* All references, tables, and figures are properly cited

* All relevant supporting information is included in the manuscript submission,

* There are no issues that prevent the paper from being properly typeset

You will receive further instructions from the production team, including instructions on how to review your proof when it is ready. Please keep in mind that we are working through a large volume of accepted articles, so please give us a few days to review your paper and let you know the next and final steps.

Lastly, if your institution or institutions have a press office, please let them know about your upcoming paper now to help maximize its impact. If they'll be preparing press materials, please inform our press team within the next 48 hours. Your manuscript will remain under strict press embargo until 2 pm Eastern Time on the date of publication. For more information, please contact onepress@plos.org.

You will receive an invoice from PLOS for your publication fee after your manuscript has reached the completed accept phase. If you receive an email requesting payment before acceptance or for any other service, this may be a phishing scheme. Learn how to identify phishing emails and protect your accounts at https://explore.plos.org/phishing.

If we can help with anything else, please email us at customercare@plos.org.

Thank you for submitting your work to PLOS ONE and supporting open access.

Kind regards,

PLOS ONE Editorial Office Staff

on behalf of

Professor Sorana D. Bolboacă

Academic Editor

PLOS One
